# Exploring the Crystal Structure Landscape of Sulfasalazine through Various Multicomponent Crystals

**DOI:** 10.1021/acs.cgd.2c01403

**Published:** 2023-07-19

**Authors:** Shan Huang, Vinay K. R. Cheemarla, Davide Tiana, Simon E. Lawrence

**Affiliations:** † School of Chemistry, Synthesis and Solid State Pharmaceutical Centre, University College Cork, Cork T12 K8AF, Ireland; ‡ Analytical and Biological Chemistry Research Facility, University College Cork, Cork T12 K8AF, Ireland

## Abstract

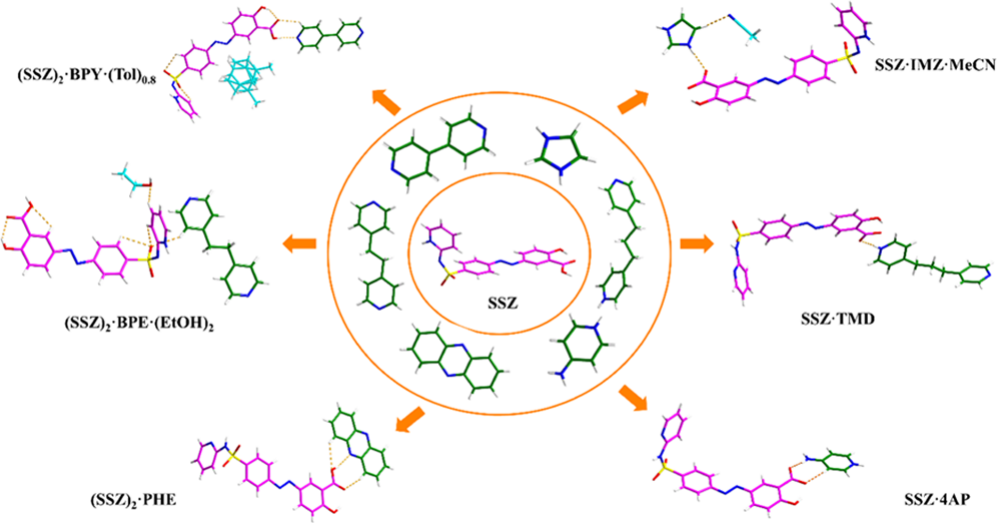

Sulfasalazine is used as an anti-inflammatory drug to treat large intestine diseases and atrophic arthritis. In the solid state, two tautomers are known: an amide tautomer (triclinic polymorph) and an imide tautomer (monoclinic polymorph). Crystallization of six new multicomponent solids of sulfasalazine with three cocrystal formers and three salt formers has been achieved by slurry, liquid-assisted grinding and slow evaporation methods. All of the solid forms are characterized by X-ray diffraction techniques, thermal analysis, and Fourier transform infrared spectroscopy. The crystal structural analysis reveals that two sulfasalazine molecules or anions arrange in a head-to-head fashion involving their pyridyl, amide, and sulfonyl groups in an *R*_2_^2^(7):*R*_2_^2^(8):*R*_2_^2^(7) motif. This is the key structural unit appearing in both sulfasalazine imide polymorph and all six multicomponent crystals. In addition, sulfasalazine exists in the amide form in all unsolvated multicomponent crystals obtained in this work and adopts the imide tautomer in the solvated cocrystals and salt. Hirshfeld surface analysis and the associated two-dimensional (2D) fingerprint plots demonstrate that sulfasalazine has significant hydrogen bond donor capability when cocrystallized and is a significant hydrogen bond acceptor in the salts. The frontier molecular orbital analysis indicates that sulfasalazine cocrystals are chemically more stable than the salts.

## Abstract 2

The key structural unit in three cocrystals and three salts of sulfasalazine is a head-to-head orientation of the sulfasalazine molecules along a short axis of the molecule involving the pyridyl and sulfonamide groups in an *R*_2_^2^(7):*R*_2_^2^(8):*R*_2_^2^(7) motif. Hirshfeld surface and frontier molecular orbital analyses investigated the differences between the cocrystals and salts.

## Introduction

**Figure 1:**
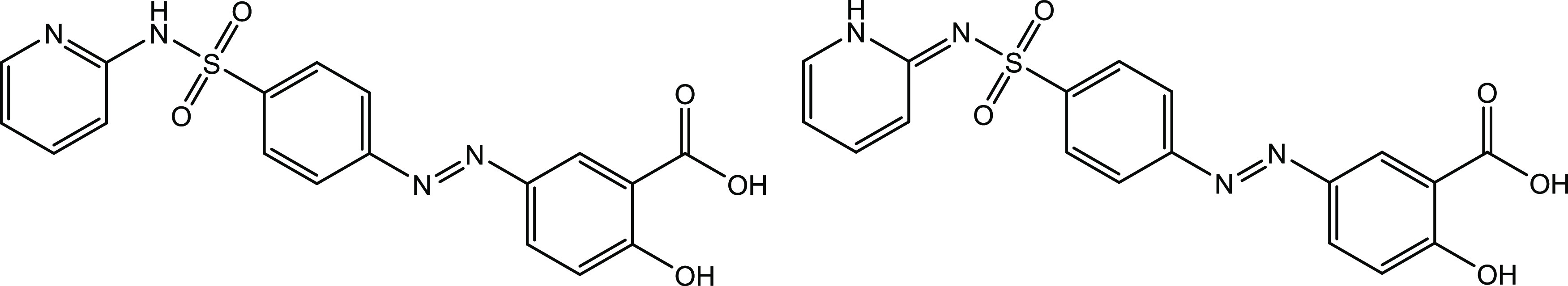
Amide form of sulfasalazine observed in the triclinic polymorph (left) and the imide form seen in the monoclinic polymorph (right).

Sulfasalazine (SSZ, Figure 1), a conjugate of an anti-inflammatory drug, 5-aminosalicylic acid, and an antibacterial drug, sulfapyridine, is successfully used as a disease-modifying anti-rheumatic drug to treat large intestine diseases and atrophic arthritis.^1^ As one of the sulfonamide compounds containing a pyridine or pyrimidine moiety, SSZ can adopt two different tautomeric conformations in its crystal forms. The first reported tautomer of SSZ was the triclinic amide form, which was obtained from ethanol by recrystallization,^2^ while the monoclinic imide tautomer was obtained by cooling after heating an ethanolic solution of SSZ in a Teflon-lined stainless steel autoclave.^3^ The migration of a hydrogen atom, accompanied by the switch of a single bond and adjacent double bond, significantly alters the crystal packing and intermolecular interactions of these two tautomers.

**Figure 2:**
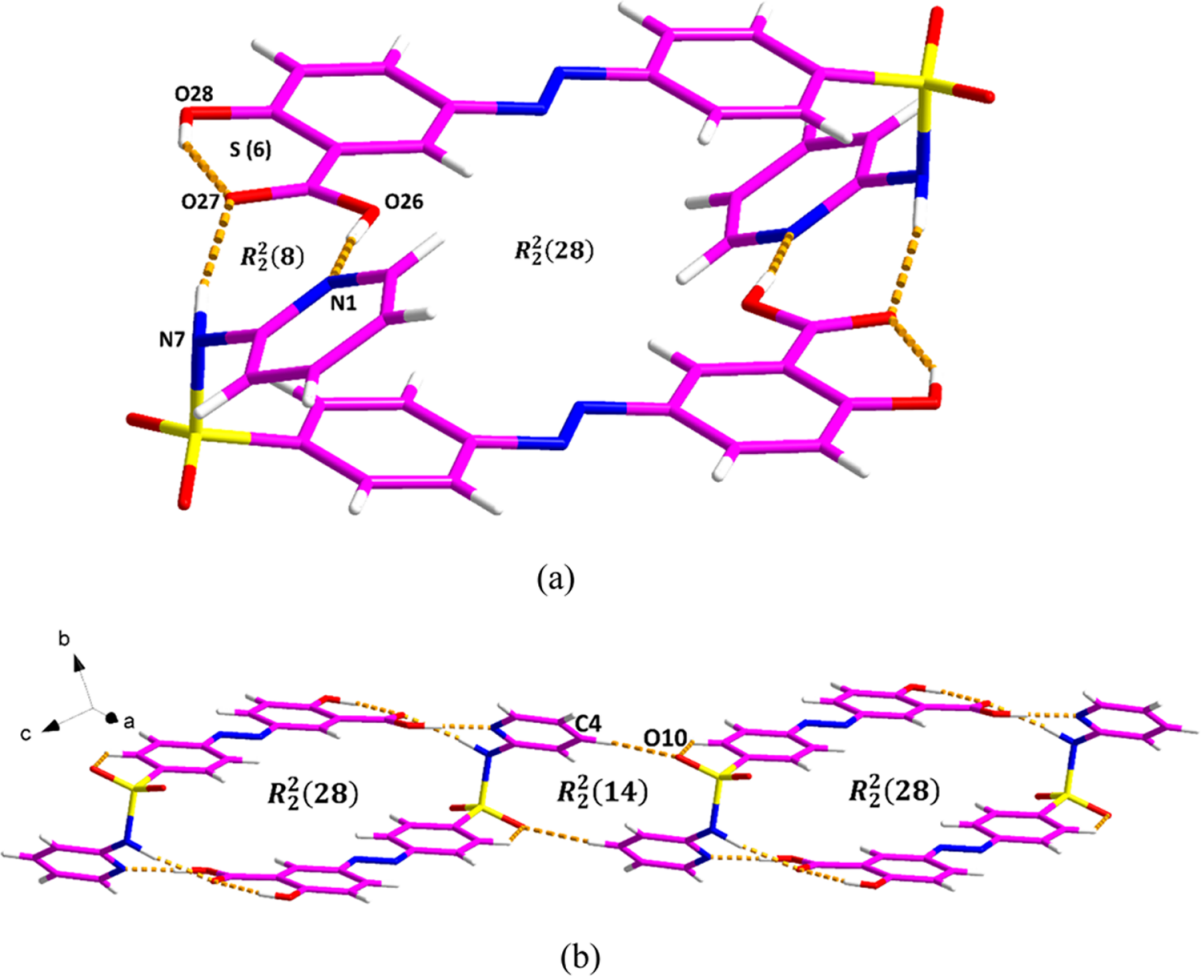
Crystal packing and intermolecular interactions in the SSZ amide form: (a) *R*_2_^2^(8) and *R*_2_^2^(28) motifs and (b) *R*_2_^2^(14) motif.

To further investigate the molecular arrangements and hydrogen bonding motifs in different SSZ crystal forms, a detailed analysis of the crystal structures of the known SSZ amide and imide forms was conducted. The hydrogen bond network and π–π interactions of the amide polymorph are shown in Figures 2 and S6a, respectively, and the corresponding hydrogen bond and π–π interaction data are displayed in Table S2. Two SSZ molecules are assembled in a head-to-tail fashion through discrete N–H···O and O–H···N hydrogen bonds (taking the pyridyl group as the head and the carboxyl acid group as the tail), generating binary level *R*_2_^2^(8) and *R*_2_^2^(28) motifs (Figure 2a). The structure is extended through C–H···O discrete hydrogen bonds, forming an *R*_2_^2^(14) motif (Figure 2b).

**Figure 3:**
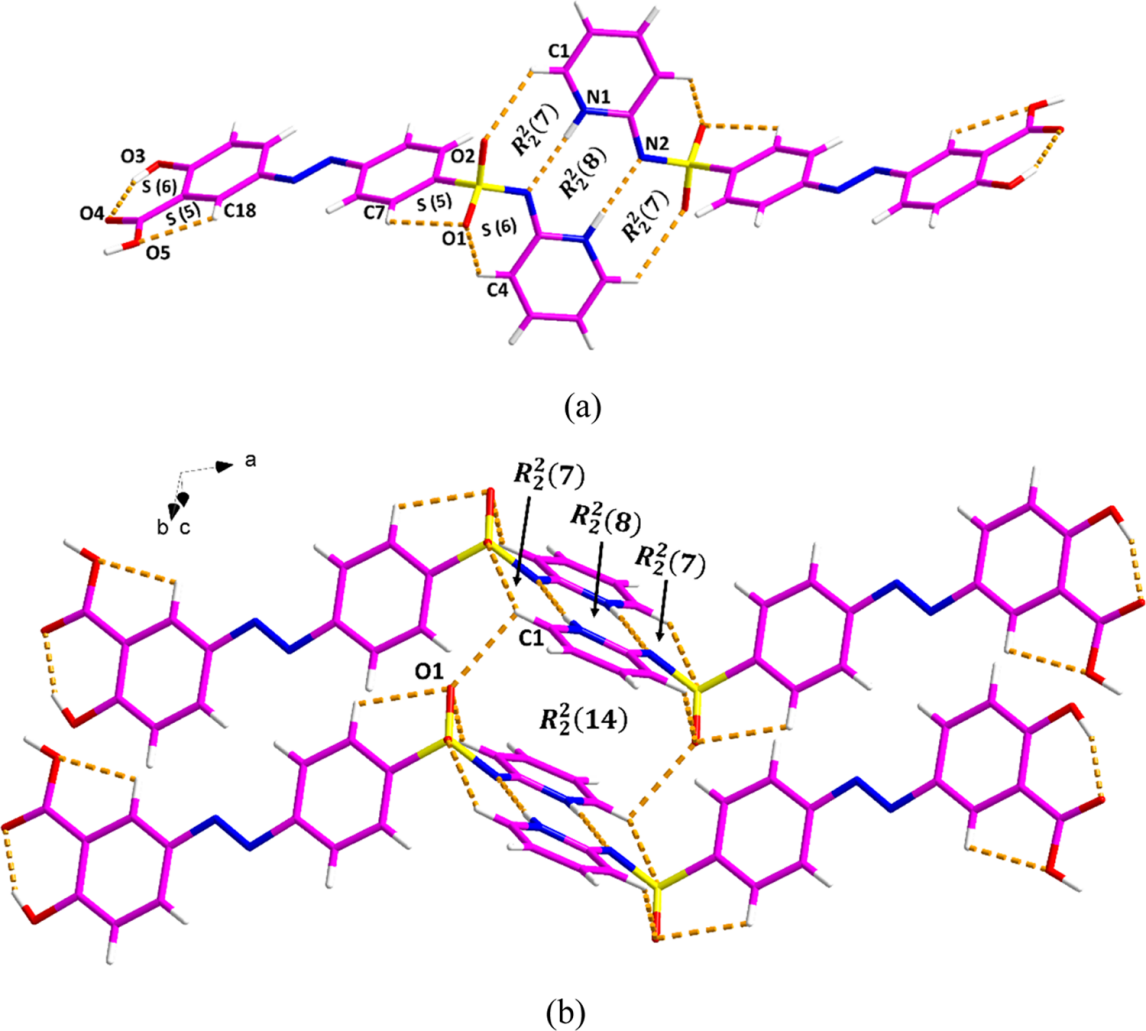
Crystal packing and intermolecular interactions in the SSZ monoclinic imide form: (a) *R*_2_^2^(7):*R*_2_^2^(8):*R*_2_^2^(7) motif and (b) *R*_2_^2^(14) motif.

In contrast, in the structure of the imide form, pairs of SSZ molecules are arranged in a head-to-head manner (Figure 3 and Table S3). The two SSZ molecules are linked via a N–H···N discrete hydrogen bond and a C–H···O discrete hydrogen bond, generating an *R*_2_^2^(7):*R*_2_^2^(8):*R*_2_^2^(7) motif (Figure 3a). Additional C–H···O discrete hydrogen bond forms an *R*_2_^2^(14) motif (Figure 3b). Furthermore, the π–π interactions between two pyridyl rings from SSZ also contribute to the extended crystal packing (Figure S7b).

Sulfasalazine has low solubility and permeability and is a class IV drug^4^ according to the Biopharmaceutics Classification System.^5^ Different approaches have been investigated to improve its bioavailability. For example, Shadid et al. successfully improved the solubility and bioavailability of SSZ by ionic liquid formation.^6^ Priyam and co-workers synthesized an amphiphilic derivative of SSZ to modify the solubility by conjugating it with polyethylene glycol.^7^ In addition, nanocrystallization,^8^ solid dispersion,^9^ and noisome techniques^10^ have been explored to improve the solubility and/or dissolution performance of SSZ.

During the past few decades, crystal engineering has developed for predicting and designing the crystals that contain more than one molecule, for example, cocrystals and salts.^11−14^ A significant driver has been the design of improved drugs with optimal physicochemical properties. Cocrystals are multicomponent crystalline materials of two or more different molecular and/or ionic compounds in a stoichiometric ratio that are neither solvates nor simple salts.^15^ They are generally assembled via hydrogen bonds,^16^ halogen bonds,^17^ or π–π stacking.^18^ The term pharmaceutical cocrystal has been used when at least one component is an active pharmaceutical ingredient and the others are pharmaceutically acceptable.^19^ Similarly, the term pharmaceutical salt has been used for related systems where intermolecular proton transfer has occurred between complementary acid and basic functional groups.^20^ Empirically, when Δp*K*_a_ [Δp*K*_a_ = p*K*_a_ (base) – p*K*_a_ (acid)] is greater than 4, the two components form a salt, and when Δp*K*_a_ is less than −1, the system results in a cocrystal. For systems with Δp*K*_a_ between −1 and 4, a linear relationship between Δp*K*_a_ and the probability of proton transfer between two components was derived.^21,22^ Other techniques are used for distinguishing crystal forms, for instance, solid-state nuclear magnetic resonance,^23^ vibrational spectroscopy,^24^ and single crystal X-ray diffraction (SCXRD).^25^

From the crystal engineering perspective, SSZ would be expected to readily form cocrystals or salts because it has multiple functional groups, with multiple hydrogen bond donor and acceptor sites. Four multicomponent crystalline materials of SSZ have been reported: SSZ-trimethoprim (one cocrystal and one salt), SSZ-nicotinamide cocrystal, and SSZ-theobromine cocrystal. They all have enhanced dissolution performance compared with pure SSZ;^26,27^ however, none of their crystal structures are available in version 2022.2.0 of the Cambridge Structural Database (CSD).^28^

**Figure 4:**
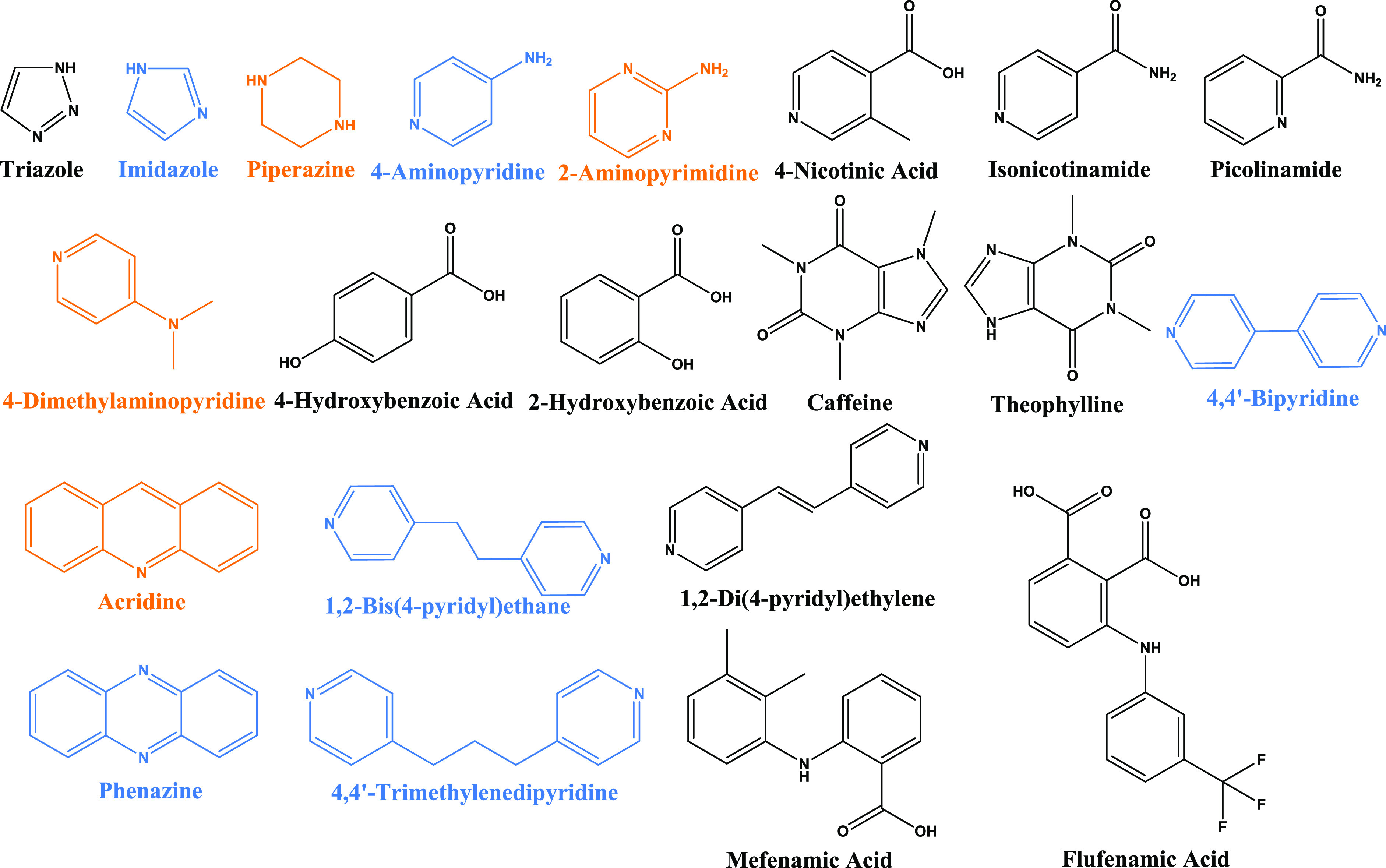
Cocrystal formers and salt formers investigated in this study (successful cocrystal/salt formers analyzed by SCXRD in blue, successful cocrystal/salt formers based on PXRD in orange, and unsuccessful cocrystal/salt formers in black).

Herein, the crystalline form diversity of SSZ with a series of pharmaceutically relevant cocrystal/salt formers (Figure 4) was explored. Three cocrystals of SSZ with 4,4′-bipyridine (BPY), 1,2-bis(4-pyridyl) ethane (BPE), and phenazine (PHE) and three salts of SSZ with 4-aminopyridine (4AP), 4,4′-trimethylenedipyridine (TMD), and imidazole (IMZ) were successfully prepared and fully characterized, and their crystal structures were obtained. Four more products of SSZ with piperazine (PPZ), 2-aminopyrimidine (2-APM), 4-dimethylaminopyridine (4-DMP), and acridine (ACRI) were determined as new multicomponent crystalline solids by powder X-ray diffraction (PXRD) (Figure S5). However, multiple attempts to grow suitable crystals for analysis were unsuccessful.

## Experimental Section

### Materials

Sulfasalazine (monoclinic imide form) was purchased from Fluorochem and used as received without further purification. Isonicotinamide, mefenamic acid, and picolinamide were purchased from TCI chemicals; other cocrystal/salt formers were obtained from Sigma-Aldrich and used as received. Solvents were purchased from commercial sources and used as received.

### Solid Form Screening and Crystallization Experiments

#### Liquid-Assisted Grinding (LAG) Method

Mechanical grinding experiments were conducted in a Retsch Mixer Mill MM 400, equipped with 5 mL stainless steel grinding jars and one 2.5 mm stainless steel grinding ball per jar. The mill was operated at a rate of 30 Hz for 30 min, and the ratio of 1:1/1:2/2:1 of SSZ with cocrystal/salt formers was used. The powdered products were isolated and analyzed by PXRD.^12^ Experimental details are provided in Table S1.

#### Slurry Method

SSZ and cocrystal/salt formers in a 1:1/1:2/2:1 molar ratio were slurried in methanol or ethanol for 2–3 days. The resulting suspension was filtered and allowed to dry in the fume hood for up to 24 h. The powdered products were isolated and analyzed by PXRD. Experimental details are provided in Table S1.

### Solution Crystallization

#### (SSZ)_2_·BPY·(Tol)_0.8_ Cocrystal Solvate

The powdered product from the 2:1 SSZ: BPY slurry experiment (48.6 mg) was dissolved in 20 mL of toluene–ethanol (1:1, v/v) in a sample vial, covered with perforated parafilm, and kept at room temperature until the solvent had almost completely evaporated (∼1 week). Red plate-like single crystals were obtained.

#### (SSZ)_2_·BPE·(EtOH)_2_ Cocrystal Solvate

The powdered (SSZ)_2_·BPE·(EtOH)_2_ (51.1 mg) obtained from slurry experiments was dissolved in 10 mL of ethanol in a sample vial, covered with perforated parafilm, and kept at room temperature until the solvent had almost completely evaporated (1–2 weeks). Orange plate-like single crystals were obtained.

#### (SSZ)_2_·PHE Cocrystal

The powdered (SSZ)_2_·PHE (50.8 mg) obtained from slurry experiments was dissolved in 10 mL of ethanol in a sample vial, covered with perforated parafilm, and kept at room temperature until the solvent had almost completely evaporated (1–2 weeks). Orange needle-like single crystals were obtained.

#### SSZ·4AP Salt

The powdered SSZ·4AP (49.6 mg) obtained from slurry experiments was dissolved in 5 mL of methanol in a sample vial, covered with perforated parafilm, and kept at room temperature until the solvent had almost completely evaporated (∼1 week). Red plate-like single crystals were obtained.

#### SSZ·TMD Salt

SSZ (19.9 mg, 0.05 mmol) and TMD (9.9 mg, 0.05 mmol) in a 1:1 molar ratio were dissolved in 10 mL of deionized water in a sample vial and left at room temperature until the solvent had almost completely evaporated (3–4 months). The orange plate-like single crystals of SSZ·TMD salt were obtained on one occasion. Attempts to obtain other crystals or bulk material were unsuccessful.

#### SSZ·IMZ·MeCN Salt Solvate

The powdered product from the 1:1 SSZ: IMZ LAG experiment (49.2 mg) was dissolved in 15 mL of acetonitrile in a sample vial, covered with perforated parafilm, and kept at room temperature until the solvent had almost completely evaporated (1–2 weeks). Orange needle-like single crystals were obtained.

### Physical Measurements

Differential scanning calorimetry (DSC) data were collected using a TA Instruments Q1000. Samples (2–6 mg) were crimped in nonhermetic aluminum pans and scanned from 25 to 300 °C at a heating rate of 10 °C min^–1^ under a continuously purged dry nitrogen atmosphere. Thermogravimetric analysis (TGA) data were collected using a TA Instruments Q500 thermogravimetric analyzer. The sample was placed in an aluminum sample pan and heated under nitrogen at a rate of 20 °C min^–1^ from 25 to 500 °C. IR spectra were recorded on a PerkinElmer UATR Two spectrophotometer using a diamond-attenuated total reflectance accessory over a range of 400–4000 cm^–1^. An average of four scans was taken for each spectrum obtained with a resolution of 4 cm^–1^. PXRD data were collected using a STOE STADI MP diffractometer with Cu Kα radiation using a linear position-sensitive detector over the 2θ range of 3.5–45.5° with an increment of 0.05° at a rate of 2° min^–1^. The samples were prepared as transmission foils and the data were viewed via STOE WinXPOW POWDAT software.^29^ SCXRD data of (SSZ)_2_·BPY·(Tol)_0.8_ and (SSZ)_2_·BPE·(EtOH)_2_ were collected using a Bruker APEX II DUO with monochromated Cu Kα radiation (λ = 1.54178 Å). SCXRD data of the other SSZ cocrystals and salts were collected on a Bruker Quest D8 diffractometer with monochromated Cu Kα radiation (λ = 1.54184 Å). All calculations and refinements were made using Bruker APEX software with the SHELX suite of programs.^30,31^ Nonhydrogen atoms were refined anisotropically. For (SSZ)_2_·BPY·(Tol)_0.8_ and (SSZ)_2_·BPE·(EtOH)_2_, the N–H hydrogen atoms were located and refined. All other hydrogen atoms were placed in geometrically calculated positions using the riding model, with C–H = 0.93–0.97 Å and N–H = 0.86–0.89 Å, and Uiso (H) (in the range of 1.2–1.5 times Ueq of the parent atom). DIAMOND was used for creating figures,^32^ and PLATON was used for the analysis of potential hydrogen bonds and short ring interactions.^33,34^ Crystallographic parameters are provided in Table 1.

**Table 1:** Crystallographic Data for SSZ Cocrystals and Salts

crystallographic data	(SSZ)_2_·BPY·(Tol)_0.8_	(SSZ)_2_·BPE·(EtOH)_2_	(SSZ)_2_·PHE	SSZ·4AP	SSZ·TMD	SSZ·IMZ·MeCN
chemical formula	C_51.6_H_42.4_N_10_O_10_S_2_	C_26_H_26_N_5_O_6_S	C_24_H_18_N_5_O_5_S	C_23_H_20_N_6_O_5_S	C_31_H_28_N_6_O_5_S	C_23_H_21_N_7_O_5_S
formula weight	1026.67	536.58	488.49	492.51	596.65	507.53
crystal system	monoclinic	triclinic	monoclinic	triclinic	triclinic	monoclinic
space group, *Z*	*P*2/*n*, 2	*P*1̅, 2	**C**2/*c*, 8	*P*1̅, 4	*P*1̅, 2	**P**2_1_/*c*, 4
temperature (K)	296.(2)	296.(2)	293(2)	293(2)	298(2)	302(2)
*a* (Å)	14.2397(5)	8.7750(7)	7.683(5)	10.7519(17)	7.826(13)	25.134(3)
*b* (Å)	6.4706(2)	12.8102(10)	33.62(3)	15.5563(18)	13.715(17)	10.0917(14)
*c* (Å)	26.7698(9)	12.9300(10)	22.610(9)	15.7326(17)	15.243(18)	9.409(2)
α (deg)	90	68.159(3)	90	64.838(7)	64.54(10)	90
β (deg)	90.5000(10)	77.234(4)	98.78(2)	74.725(10)	81.12(7)	91.511(12)
γ (deg)	90	82.571(4)	90	87.389(10)	75.13(12)	90
volume (Å^3^)	2466.46(14)	1313.99(18)	5771(6)	2290.9(5)	1426(4)	2385.8(7)
ρ calcd (g cm^–3^)	1.382	1.356	1.124	1.428	1.390	1.413
μ (mm^–1^)	1.571	1.524	1.320	1.678	1.449	1.640
reflns measured	30288	16382	20087	73865	11530	54017
reflns independent	4321	4409	2835	8952	6638	4714
*R*_int_	0.0220	0.0201	0.0491	0.0887	0.0738	0.0629
significant [*I* > 2σ(*I*)]	4140	3996	2277	5947	1163	4055
parameters refined	338	366	319	628	390	328
Δρ_max_, Δρ_min_ (e Å^–3^)	0.484, −0.355	0.508, −0.422	0.198, −0.300	1.050, −0.526	0.137, −0.204	0.508, −0.287
*F*(000)	1068	562	2024	1024	624	1056
*R*_1_ [*I* > 2σ(*I*)]	0.0473	0.0673	0.0512	0.0872	0.0593	0.0573
w**R**_2_ (all data)	0.1492	0.1988	0.2007	0.2842	0.1647	0.1829
CCDC	2109809	2109810	2109811	2064484	2109807	2109808

### Computational Studies

Density functional theory (DFT) calculations using the Gaussian 09 program package employing the M06-2X functional with the 6-31+G (d,p) basis set were performed on the six obtained crystals without conducting structural optimization.^13,35^ The molecular orbitals were viewed using the Multiwfn 3.8 program and plotted by VMD.^36,37^ Hirshfeld surface analysis and two-dimensional (2D) fingerprint plots were carried out using the CrystalExplorer 21.5 program.^38^

## Results and Discussion

### Physical Characterization

The thermal behavior of the SSZ cocrystals/salts was assessed using DSC and TGA techniques. The melting points of (SSZ)_2_·BPY·(Tol)_0.8_, (SSZ)_2_·BPE·(EtOH)_2_, SSZ·4AP, SSZ·TMD, and SSZ·IMZ·MeCN are in between those of the individual components, while (SSZ)_2_·PHE cocrystal melts at a lower temperature than the starting materials (Figure S1). Additionally, small endothermic peaks before the melting peaks were observed in the DSC traces for (SSZ)_2_·BPY·(Tol)_0.8_, (SSZ)_2_·BPE·(EtOH)_2_, and SSZ·IMZ·MeCN, indicating the presence of solvent within the crystal lattice, which is consistent with the SCXRD data. This is also supported by the TGA results (Figure S2). A weight loss of 6.8% is observed for (SSZ)_2_·BPY·(Tol)_0.8_, which corresponds to 0.8 equiv of toluene (calculated as 6.7%). For (SSZ)_2_·BPE·(EtOH)_2_, a weight loss of 8.7%, corresponding to 1 equiv of EtOH (calculated as 8.6%), is observed. Similarly, SSZ·IMZ·MeCN exhibits a significant weight loss of 7.8%, which corresponds to 1 equiv of MeCN (calculated value is 8.1%). No significant weight loss before the decomposition temperature is observed for the other SSZ solids, suggesting that they are not solvated or hydrated. After cocrystallization of SSZ, the Fourier transform infrared (FTIR) spectrum (Figure S3) of cocrystals and salts showed the shifts in the hydroxyl peak of SSZ from 3027 to 2974 [(SSZ)_2_·BPY·(Tol)_0.8_], 2978 [(SSZ)_2_·BPE·(EtOH)_2_], 3059 [(SSZ)_2_·PHE], 2980 (SSZ·4AP), 3058 (SSZ·IMZ·MeCN), 3052 (SSZ·TMD) cm^–1^, suggesting the formation of new crystalline forms of SSZ, respectively. The PXRD patterns of the (SSZ)_2_·BPY·(Tol)_0.8_, (SSZ)_2_·BPE·(EtOH)_2_, (SSZ)_2_·PHE, SSZ·4AP, and SSZ·IMZ·MeCN (Figure S4) matched with the theoretical patterns obtained from the SCXRD analysis, demonstrating that these cocrystals can be reproduced in bulk quantities by the slurry or LAG method. The PXRD pattern of SSZ-TMD cannot be obtained since attempts to synthesize crystals or bulk material of SSZ·TMD were unsuccessful.

As for the solid states of these obtained new crystals, according to the Δp*K*_a_ rule, (SSZ)_2_·PHE is expected to be a cocrystal (Δp*K*_a_ <−1), while SSZ·4AP and SSZ·IMZ·MeCN are expected to be salts (Δp*K*_a_ > 4), which are confirmed by the SCXRD results (Table 2). The solid state of (SSZ)_2_·BPY·(Tol)_0.8_, (SSZ)_2_·BPE·(EtOH)_2_, and SSZ·TMD could be either salt or cocrystal (−1 < Δp*K*_a_ < 4), and SCXRD data confirm that (SSZ)_2_·BPY·(Tol)_0.8_ and (SSZ)_2_·BPE·(EtOH)_2_ are cocrystals and SSZ·TMD is a salt, which follows the linear relationship proposed by Cruz-Cabeza.^22^

**Table 2:** p*K*_a_ Values of SSZ, Cocrystal/Salt Formers, and Δp*K*_a_ Values of the New Solid Forms

	p*K*_a_	Δp*K*_a_	solid state
SSZ	2.70^a^		
BPY	3.27^a^	0.57	2:1 cocrystal solvate
BPE	6.13^a^	3.43	2:1 cocrystal solvate
PHE	1.60^a^	–1.10	2:1 cocrystal
4AP	9.17^39^	6.47	1:1 salt
TMD	6.30^a^	3.60	1:1 salt
IMZ	6.97^40^	4.27	1:1:1 salt solvate

a p*K*_a_ was obtained from CAS SciFinder^n^.

### Crystal Structures

The structure analyses of the six multicomponent systems are presented below. Hydrogen bond and π–π interaction data are displayed in Tables S4–S9.

#### (SSZ)_2_·BPY·(Tol)_0.8_ Cocrystal Solvate

**Figure 5:**
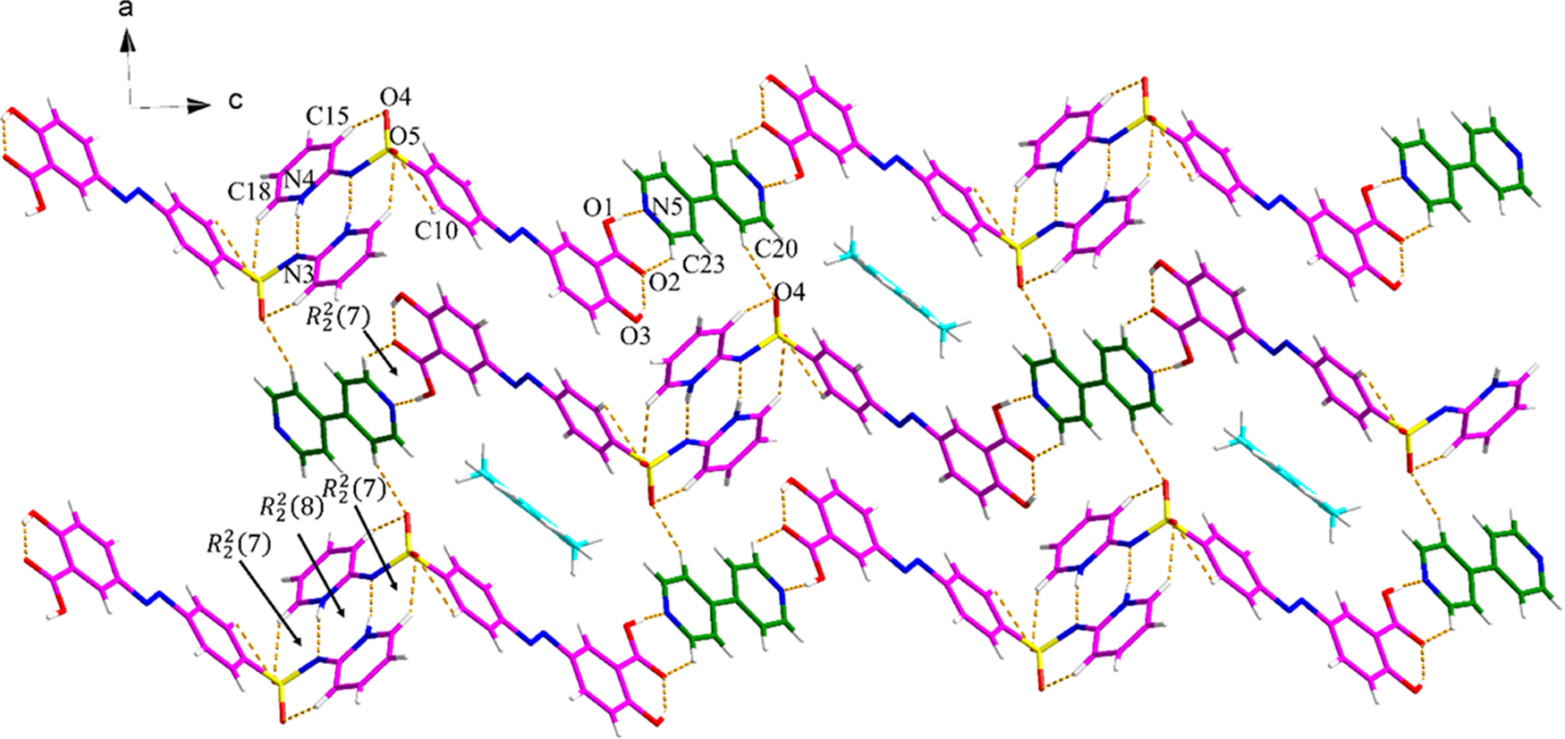
Crystal packing and intermolecular interactions in the (SSZ)_2_·BPY·(Tol)_0.8_ cocrystal solvate (pink is SSZ, green is BPY, and blue is toluene).

The (SSZ)_2_·BPY·(Tol)_0.8_ crystal has one SSZ molecule and half of the BPY molecule in the asymmetric unit. Disordered toluene is present in voids in the structure. An *R*_2_^2^(7) motif is formed by SSZ and BPY through O1–H1···N5 and C23–H23···O2 discrete hydrogen bonds, and the BPY molecule links another SSZ molecule via a discrete C20–H20···O4 hydrogen bond. The latter SSZ molecule is involved in two hydrogen bonds (N4–H4N···N3 and C18–H18···O5) with the adjacent SSZ molecule, forming an *R*_2_^2^(7):*R*_2_^2^(8):*R*_2_^2^(7) motif, which leads to the formation of three-dimensional (3D) hydrogen bond layers (Figure 5). The structure is further stabilized by the π–π interactions between the SSZ and SSZ, SSZ and BPY, and SSZ-toluene molecules (Figure S8 and Table S4).

#### (SSZ)_2_·BPE·(EtOH)_2_ Cocrystal Solvate

**Figure 6:**
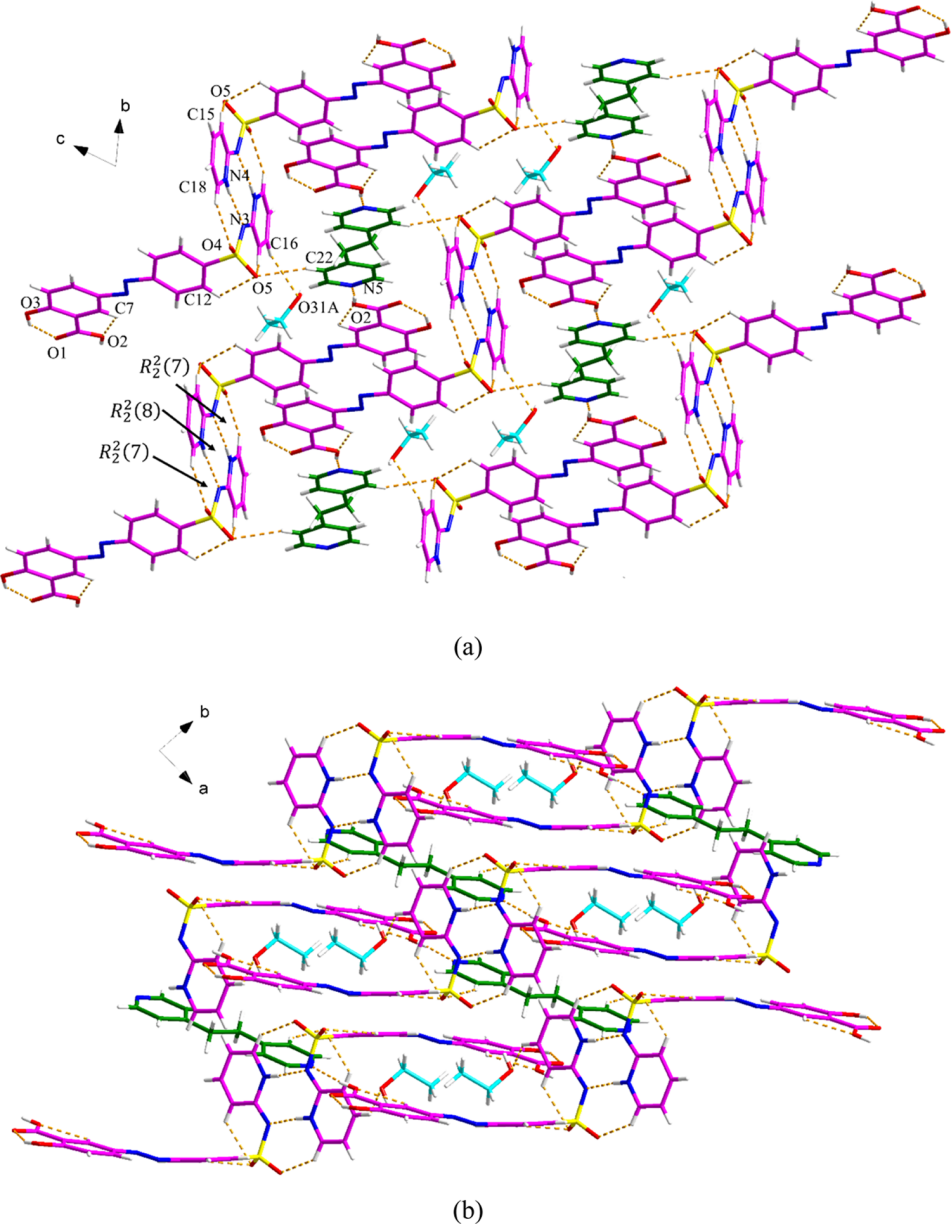
Crystal packing and intermolecular interactions in the (SSZ)_2_·BPE·(EtOH)_2_: (a) along the *a*-axis (pink is SSZ, green is BPE, and blue is EtOH) and (b) along the *c*-axis. The minor component of the disordered structure has been omitted for clarity.

The (SSZ)_2_·BPE·(EtOH)_2_ cocrystal solvate crystallizes in the triclinic P1̅ space group with one SSZ molecule, half of the BPE molecule, and one EtOH molecule in the asymmetric unit. The SSZ molecule is disordered over the N=N group (75:25), and the EtOH molecule is disordered (75:25). As shown in Figure 6a, the BPE molecule links two SSZ molecules through discrete O2–H2···N5 and C22–H22···O5 hydrogen bonds, and the EtOH molecule links one SSZ molecule via C16–H16···O31A hydrogen bonds. Along the *a*-axis, the 3D hydrogen bonding network is further stabilized by the *R*_2_^2^(7):*R*_2_^2^(8):*R*_2_^2^(7) motif between two adjacent SSZ molecules. Along the *c*-axis, the voids containing EtOH molecules can be observed (Figure 6b). Additional π–π interactions between the phenyl rings from SSZ and the pyridyl rings from BPE (Cg2–Cg3, Cg2–Cg4, and Cg4–Cg4) contribute to the extended 3D structure (Figure S9 and Table S5).

#### (SSZ)_2_·PHE Cocrystal

**Figure 7:**
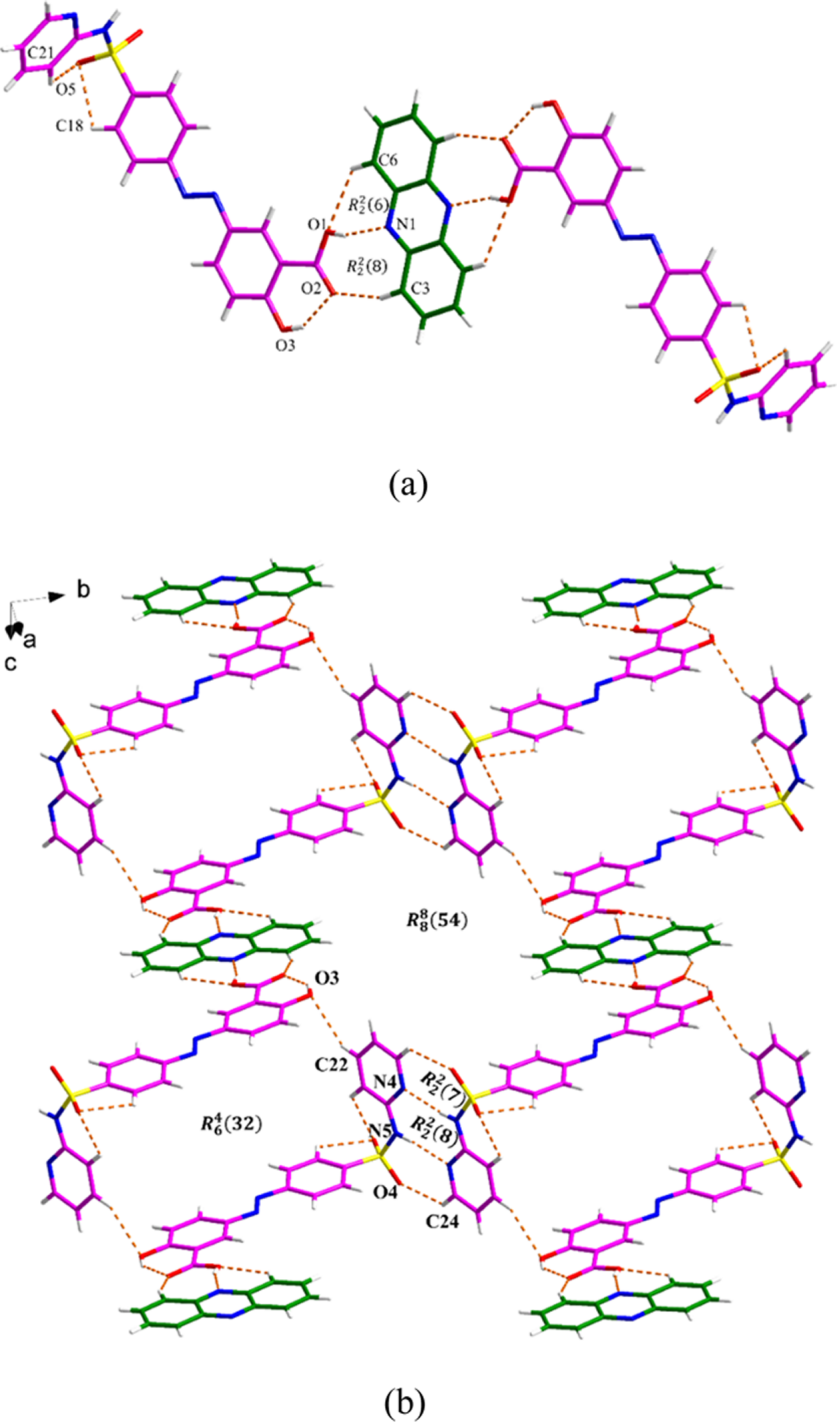
Crystal packing and intermolecular interactions in the (SSZ)_2_·PHE: (a) asymmetric unit (pink is SSZ and green is PHE) and (b) hydrogen bond network.

The asymmetric unit of (SSZ)_2_·PHE has one SSZ molecule and half of the PHE molecule. PHE and two SSZ molecules form an *R*_2_^2^(6):*R*_2_^2^(8) motif through C6–H6···O1, O1–H1···N1 and C3–H3···O2 discrete hydrogen bonds (Figure 7a). The hydrogen bond network is extended through N4–H40···N5 and C24–H24···O4 hydrogen bonds, resulting in the same *R*_2_^2^(7):*R*_2_^2^(8):*R*_2_^2^(7) motif between adjacent SSZ molecules as mentioned previously (Figure 7b). In addition, an *R*_6_^4^(32) ring is formed via the C22–H22···O3 interaction between two SSZ molecules, and an *R*_8_^8^(54) ring is formed between six SSZ molecules and two PHE molecules. The π–π interactions between two phenyl rings from SSZ and PHE (Cg2–Cg2, Cg3–Cg5) also contribute to the extended crystal packing (Figure S10 and Table S6).

#### SSZ·4AP Salt

**Figure 8:**
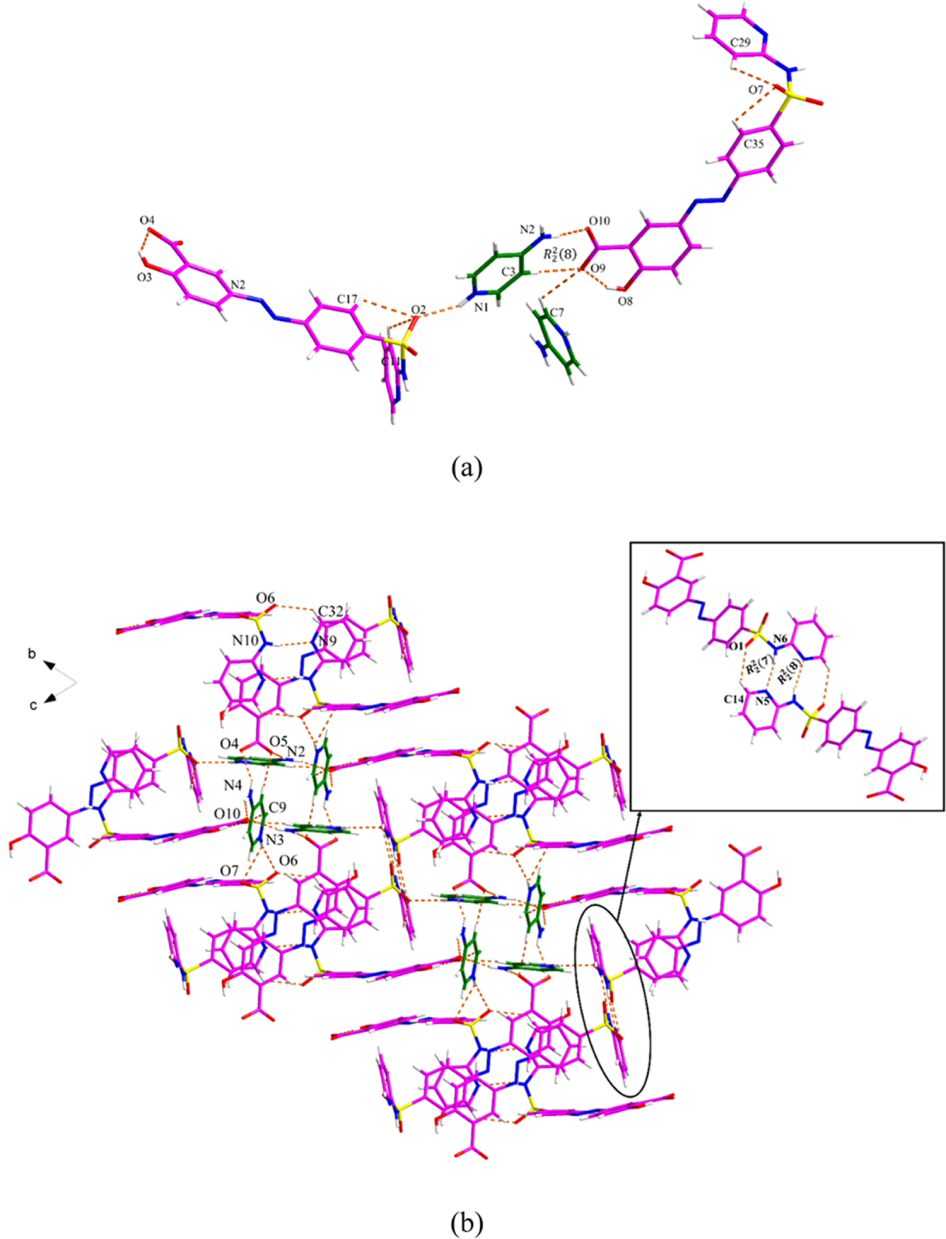
Crystal packing and intermolecular interactions in the SSZ·4AP: (a) asymmetric unit (pink is SSZ and green is 4AP) and (b) three-dimensional hydrogen bond network.

The SSZ·4AP salt crystallizes with two SSZ^–^ anions and two 4AP^+^ cations in the asymmetric unit. The proton is transferred from the carboxylic acid group of SSZ to the pyridyl ring of 4AP. SSZ^–^ and 4AP^+^ are linked through N1–H1N···O2, C7–H7···O9, N2–H2A···O10 and C3–H3···O9 discrete hydrogen bonds, the latter two forming an *R*_2_^2^(8) motif (Figure 8a). The assembly is further sustained by intermolecular interactions (C9–H9···O5, N4–H4B···O4, N2–H2B···O5, N3–H3N···O6, N4–H4A···O10, and N3–H3N···O7) between two SSZ^–^ anions and two 4AP^+^ cations in the same manner. The two adjacent SSZ^–^ interact via N–H···N and C–H···O hydrogen bonds, which constitute an *R*_2_^2^(7):*R*_2_^2^(8):*R*_2_^2^(7) motif (Figure 8b). Additional π–π stacking interactions between adjacent phenyl rings from SSZ^–^ also contribute to the extended structure (Figure S11 and Table S7).

#### SSZ·TMD Salt

**Figure 9:**
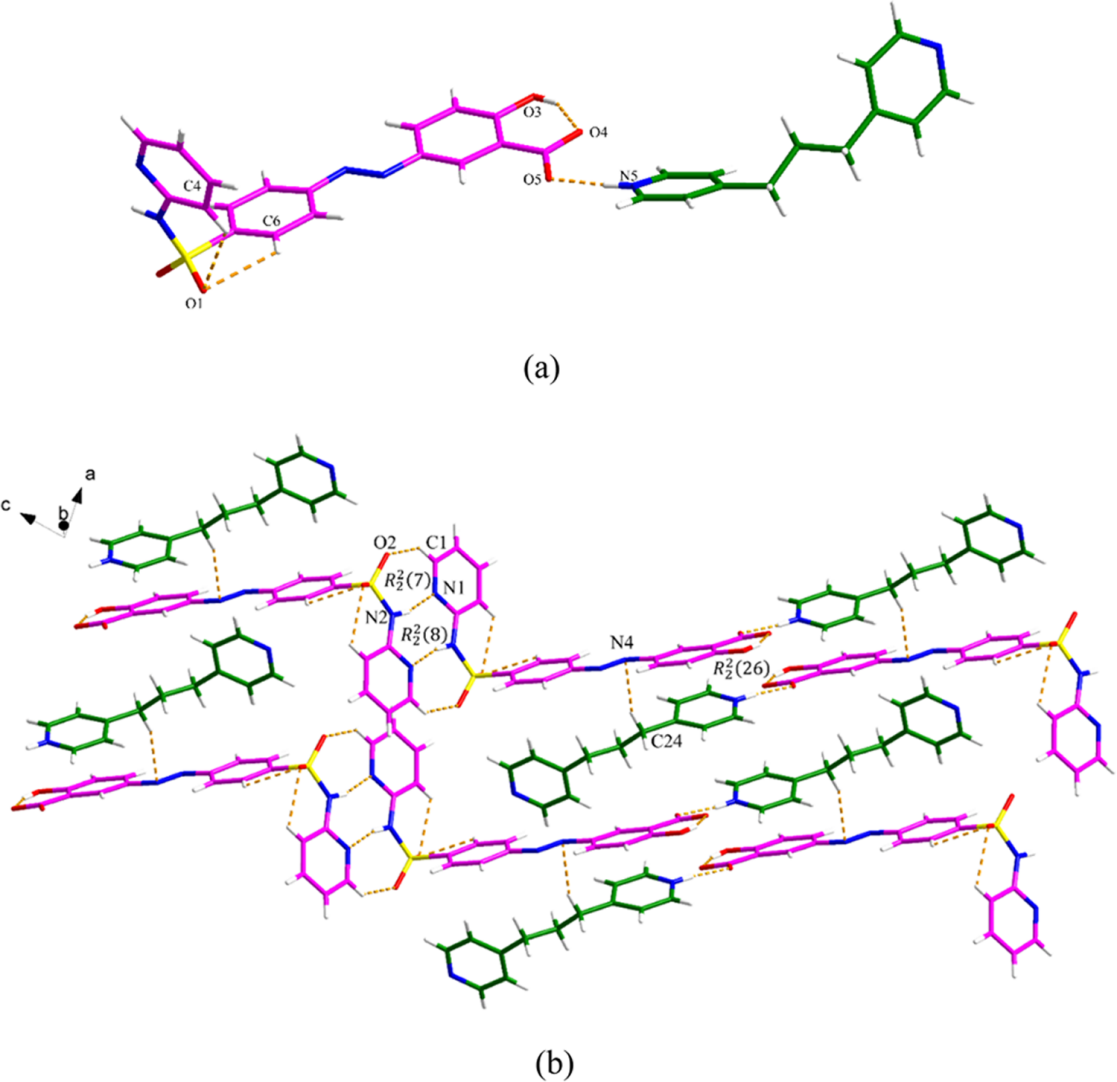
Crystal packing and intermolecular interactions in the SSZ·TMD: (a) asymmetric unit (pink is SSZ and green is TMD) and (b) hydrogen bond network.

SSZ and TMD form a salt that crystallizes with one TMD^+^ cation and one SSZ^–^ anion in the asymmetric unit. The two components interact with each other through the N5–H5···O5 discrete hydrogen bond (Figure 9a). What is interesting is that only in this crystal is the N atom of the azo group from the SSZ^–^ anion involved in the formation of intermolecular hydrogen bonds, producing an *R*_2_^2^(26) motif between two asymmetric units via the C24-H24B···N4 hydrogen bond. Two adjacent SSZ^–^ anions connect through N2–H2···N1 and C1–H1···O2 hydrogen bonds, forming the same *R*_2_^2^(7):*R*_2_^2^(8):*R*_2_^2^(7) motif as the previously described crystals (Figure 9b). As shown in Figure S12 and Table S8, the packing is further stabilized by the π–π interactions between the phenyl rings from SSZ^–^ and the pyridyl rings from TMD^+^ (Cg2–Cg3, Cg4–Cg4, and Cg5–Cg5).

#### SSZ·IMZ·MeCN Salt Solvate

**Figure 10:**
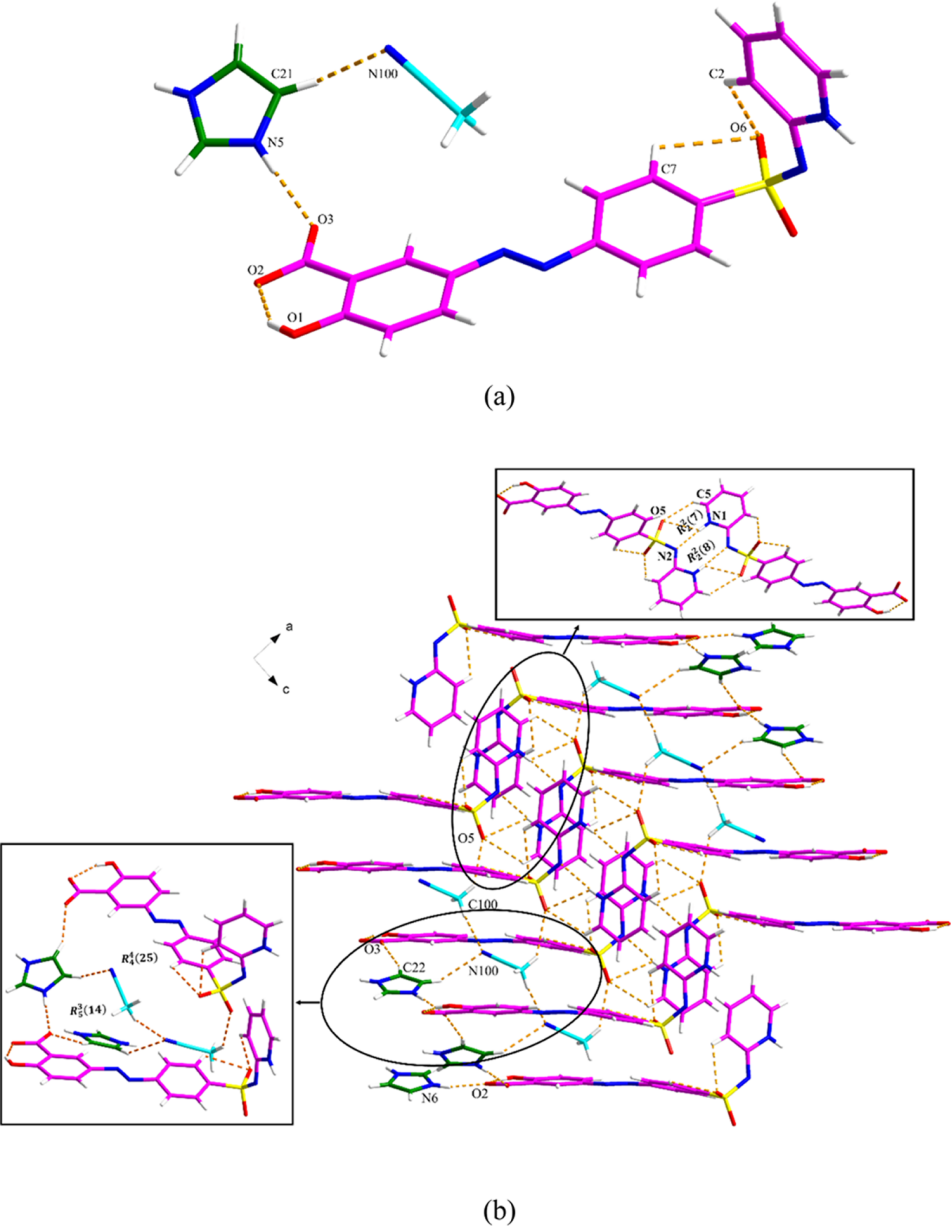
Crystal packing and intermolecular interactions in the SSZ·IMZ·MeCN: (a) asymmetric unit (pink is SSZ, green is IMZ, and blue is MeCN) and (b) hydrogen bond network.

SSZ·IMZ·MeCN crystallizes with one SSZ^–^ anion, one IMZ^+^ cation, and one acetonitrile molecule in the asymmetric unit. The proton is transferred from the carboxylic acid of SSZ^–^ to the basic nitrogen of the imidazole ring (Figure 10a). Along the *b*-axis, the IMZ^+^ cation acts as hydrogen bond donors and interacts with SSZ^–^ and MeCN through N5–H5A···O3, N6–H6···O2, and C21–H21···N100 discrete hydrogen bonds, respectively (Figure 10b). In addition, *R*_5_^3^(14) and *R*_4_^4^(25) motifs are formed via the above interactions and C100–H10B···N100, C100–H10C···O5, and C22–H22···O3 hydrogen bonds. The two adjacent SSZ^–^ anions interact with each other through C5–H5···O5 and N1–H1A···N2 hydrogen bonds, forming a related *R*_2_^2^(7):*R*_2_^2^(8):*R*_2_^2^(7) motif, which differs due to the position of the hydrogen atom. As shown in Figure S13 and Table S9, the 3D structure is further stabilized by the π–π interactions between the phenyl rings and pyridyl rings from SSZ (Cg1–Cg1 and Cg2–Cg3).

**Figure 11:**
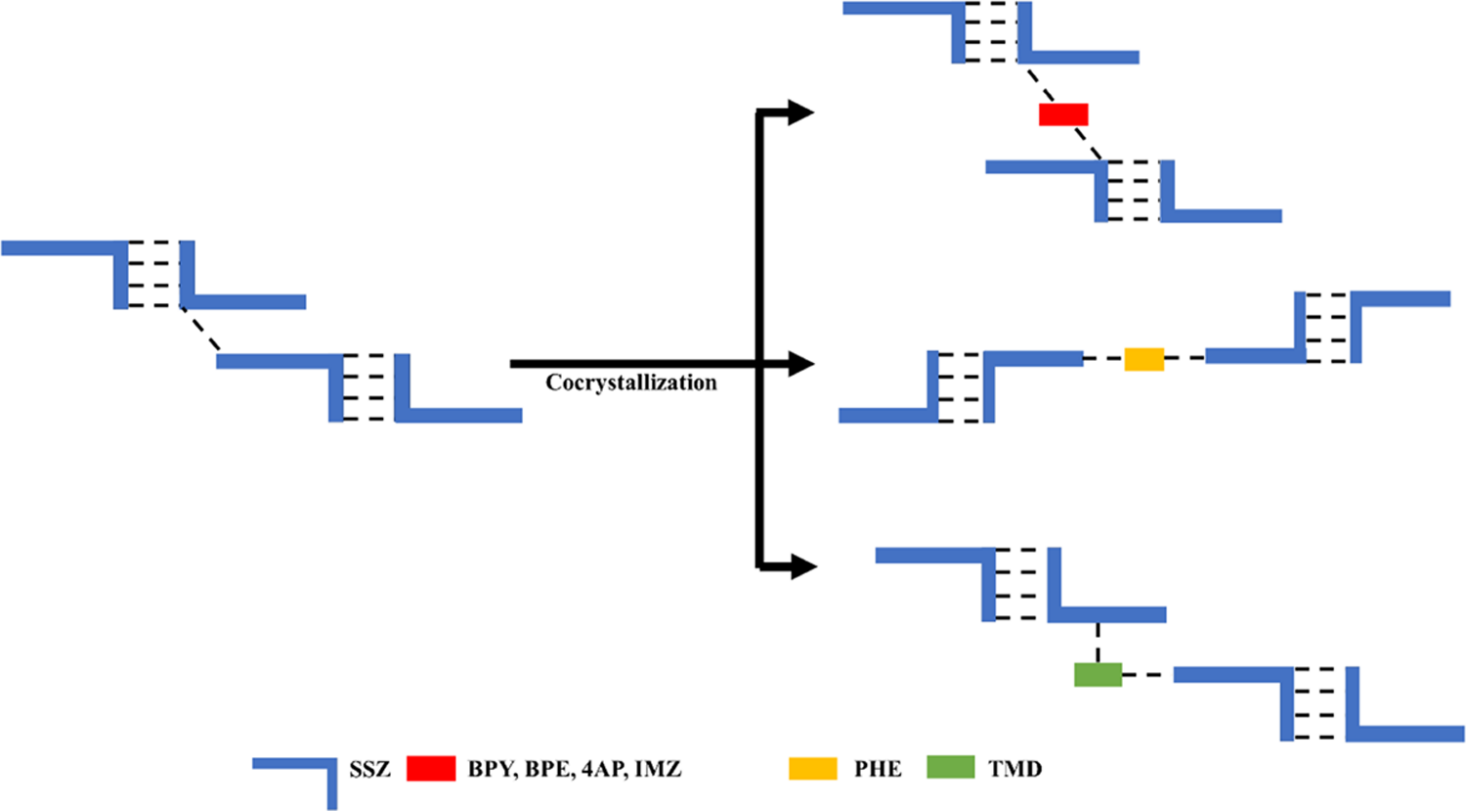
Hydrogen bonds in pure SSZ (starting material) (left) and the different arrangements with the head-to-head SSZ pairs of molecules (right).

Overall, the introduction of cocrystal/salt formers has disrupted the hydrogen bonds involving the pairs of SSZ molecules in the imide SSZ, forming six multicomponent systems with different molecular arrangements and crystal packings with the *R*_2_^2^(7):*R*_2_^2^(8):*R*_2_^2^(7) motif of pairs of SSZ molecules maintained (Figure 11).

#### Conformation of SSZ

**Figure 12:**
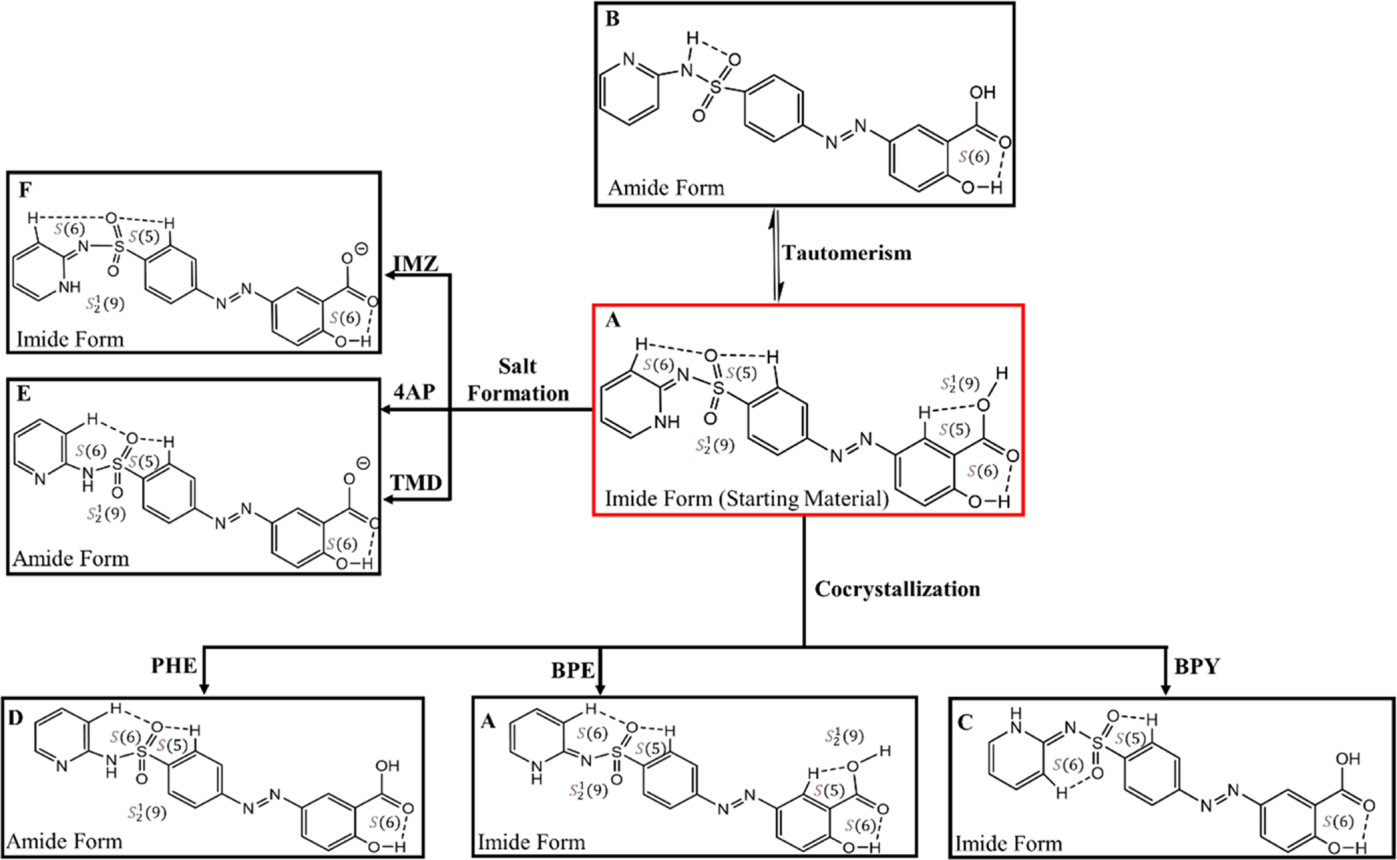
Molecular conformations of SSZ/SSZ^–^ in solid forms.

The starting material SSZ used in this work is in conformation A, where the two intramolecular hydrogen bonds (C–H···O and O–H···O) form an *S*_2_^1^(9) motif and direct the orientation of the carboxylic acid group (Figure 12). The oxygen atom of the sulfonyl group in the amide tautomer of SSZ (conformation B) is involved in one intramolecular hydrogen bonding, forming a four-membered N–S=O···H intramolecular hydrogen bond. SSZ exists in different conformations when cocrystallized with the different cocrystal/salt formers, with the amide tautomer seen in three unsolvated crystals and the imide tautomer seen in the solvated cocrystals and salt. For the (SSZ)_2_·BPY·(Tol)_0.8_ cocrystal solvate, the intramolecular hydrogen bonds C–H···O=S=O···H–C involving both oxygen atoms of the sulfonyl group lock the conformation of the SSZ molecules (conformation C), while in (SSZ)_2_·BPE·(EtOH)_2_ SSZ, the conformation is the same as the pure starting material (imide form, conformation A). In the solvated salt SSZ·IMZ·MeCN, the SSZ exists in conformation F, which only differs by the absence of the *S*(5) motif due to proton transfer. For (SSZ)_2_·PHE cocrystal (conformation D), SSZ·4AP salt and SSZ·TMD salt (conformation E), only one oxygen atom of the sulfonyl group is utilized, leading to an *S*_2_^1^(9) motif, and the carbonyl oxygen atom form has an intramolecular interaction with the hydroxyl group, creating an *S*(6) motif. The only difference between conformations D and E is whether the hydroxyl group from the carboxyl group is involved in the formation of an *S*(5) motif.

**Figure 13:**
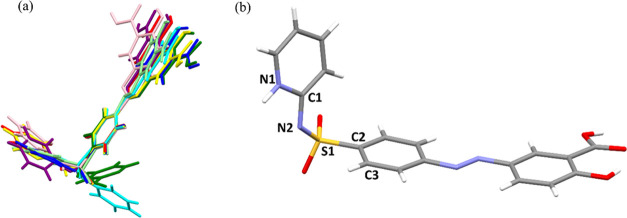
(a) Molecular overlay of SSZ: SSZ (starting material, imide form) purple, SSZ (amide form) green, (SSZ)_2_·BPY·(Tol)_0.8_ blue, (SSZ)_2_·BPE·(EtOH)_2_ pink, (SSZ)_2_·PHE yellow, SSZ·4AP light green, SSZ·TMD red, and SSZ·IMZ·MeCN cyan, and (b) selected bonds and torsions in the SSZ molecule.

Molecular overlay (Figure 13a) indicates that the bulk of the molecule (including the sulfur atom, the phenyl ring, the azo bridge, and the hydroxybenzoic acid segment) is almost planar, while the orientation of the (2-pyridylamino) sulfonyl group varies significantly. In the imide form, both C1–N2 and N2–S1 bond lengths [1.35 (4) and 1.59 (3) Å] are much shorter than those in the amide form [1.43 (2) and 1.65 (16) Å], revealing conjugation between the pyridine ring and the side chain. Moreover, the N1–C1–N2–S1 and N2–S1–C2–C3 torsion angles of the two tautomers differ.^2,3^ Regardless of the conformation of SSZ, the C1–N2 and N2–S1 bond lengths of SSZ in the six systems are closer to those of the pure imide SSZ, and no general rule in the N1–C1–N2–S1 and N2–S1–C2–C3 torsion angles can be found (Table S10).

#### Array of SSZ

**Figure 14:**
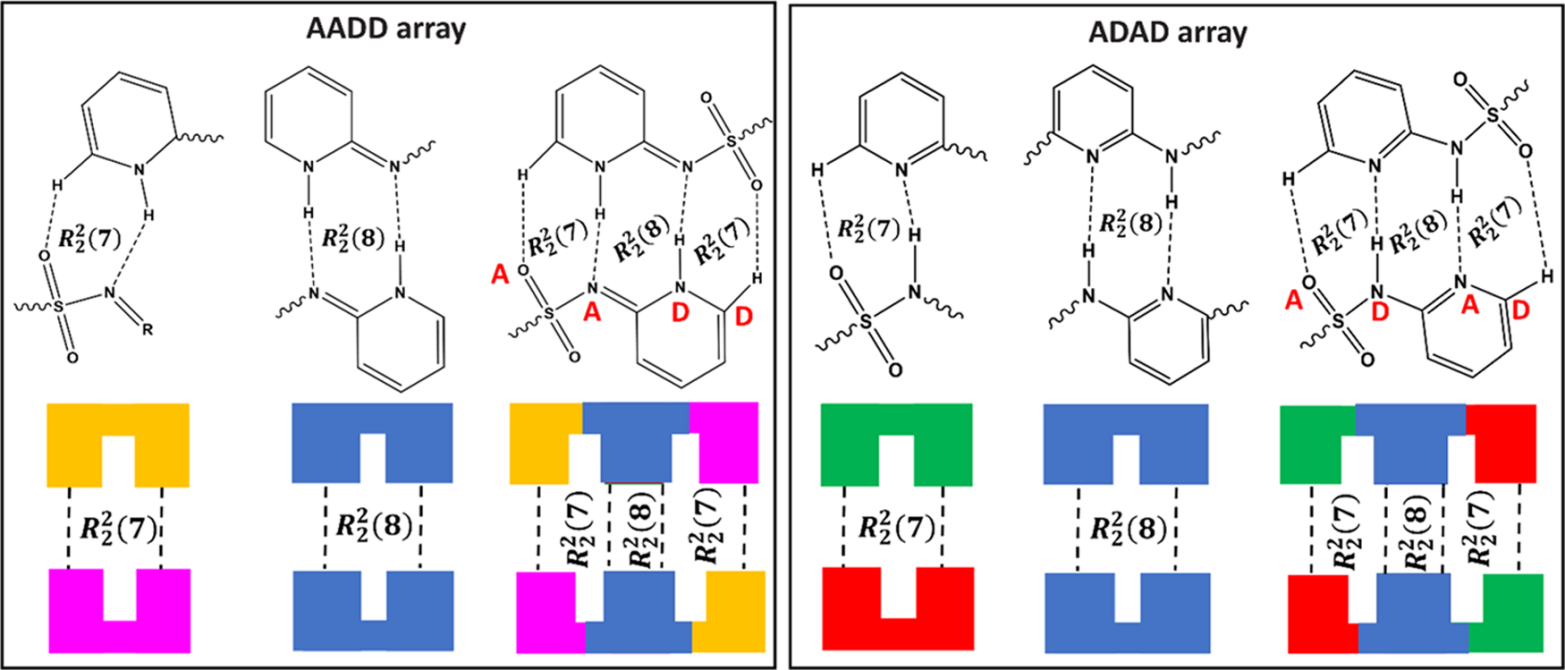
AADD/ADAD array (from the sulfonyl group to the pyridyl group) observed between two SSZ molecules/SSZ^–^ anions in SSZ solids.

There are two scenarios of the *R*_2_^2^(7):*R*_2_^2^(8):*R*_2_^2^(7) motif involving two adjacent SSZ molecules observed in the six multicomponent crystals. These arise due to the different positions of the hydrogen atom: the imide tautomer leads to the AADD array (Figure 14, left) and the amide tautomer leads to the ADAD array (D: hydrogen bond donor, A: hydrogen bond acceptor) (Figure 14, right). The three unsolvated SSZ multicomponent materials in this work have the ADAD array in their crystal structures. In contrast, the AADD array is observed in the solvated SSZ multicomponent materials, and this array is also present in the pure imide form of SSZ.^3^

**Figure 15:**
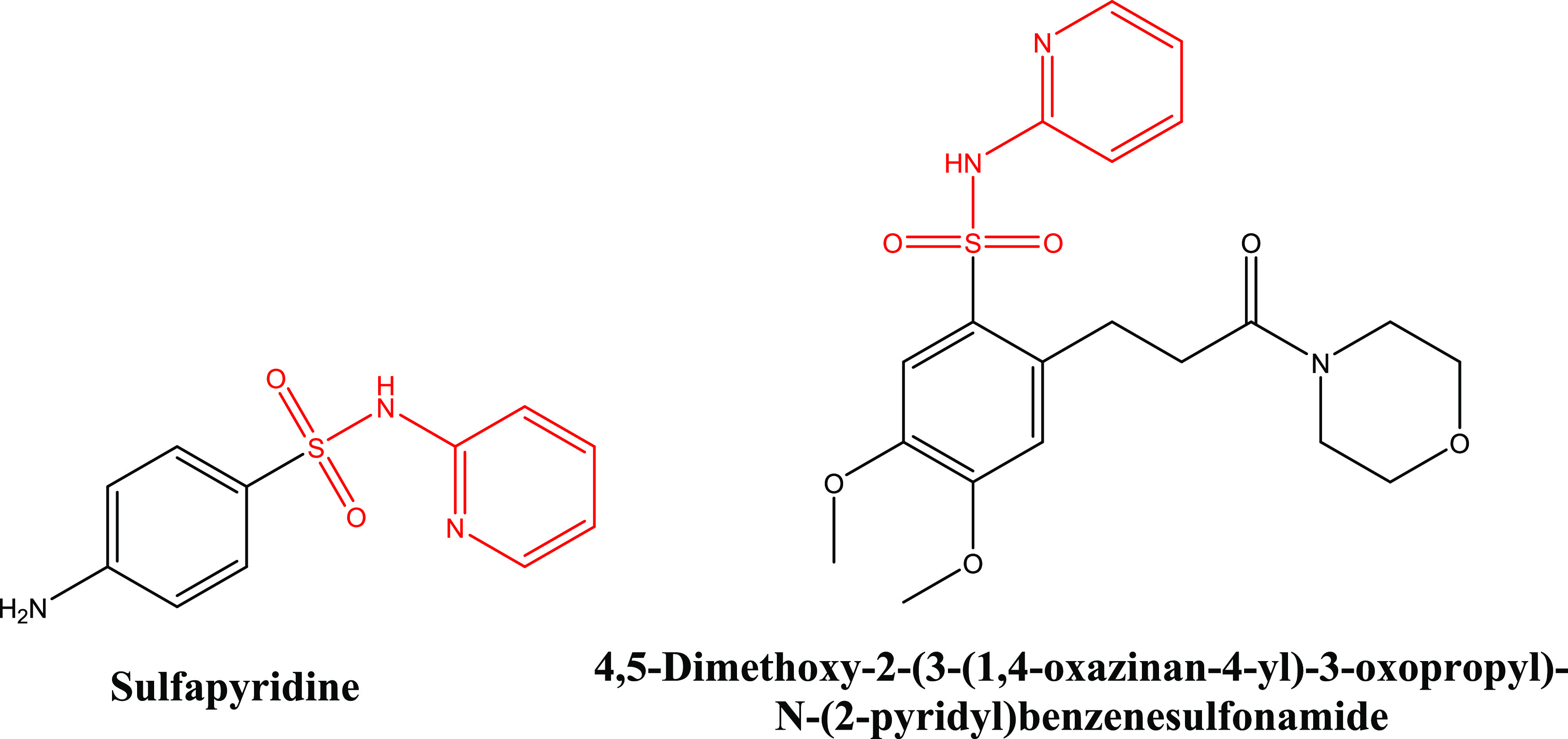
Chemical structure of sulfapyridine and 4,5-dimethoxy-2-(3-(1,4-oxazinan-4-yl)-3-oxopropyl)-*N*-(2-pyridyl)benzenesulfonamide.

To further investigate the occurrence of these two kinds of array in other multicomponent crystalline materials of sulfonamide compounds, a CSD search was conducted using ConQuest (version 2022.2.0) and the results were filtered by “3D coordinates determined,” “only single crystal structures,” and “only organics.” Both of the arrays can be found in multicomponent crystalline materials of sulfapyridine (Figure 15, left), which is not surprising since the molecular structure of sulfapyridine is a substructure of SSZ. The AADD array is found in sulfapyridine 1,3-dioxane and sulfapyridine tetrahydrofuran solvate,^41^ while the ADAD array is observed in sulfapyridine oxalic acid dibutyl ester cocrystal.^42^ In addition to this, the ADAD array also exists in three complexes of SSZ with calcium, magnesium, and strontium, and a methanol solvate of a sulfonamide compound (Figure 15, right). Therefore, it appears that the pyridine-2-amine moiety in sulfonamide compounds plays an important role in the formation of the AADD or ADAD array in the crystal structures.

### Hirshfeld Surface Analysis

Hirshfeld surfaces have proven to be a unique tool to investigate and visualize different types of intermolecular interactions in the crystal, and the 2D fingerprint plots provide quantitative information on these interactions.^43,44^ To investigate the influence of different cocrystal/salt formers on the intermolecular interactions of SSZ in different cocrystals/salts, the Hirshfeld surface analysis was performed using the CrystalExplorer 21.5 program. Figures S14 and 16 illustrate the Hirshfeld surfaces of SSZ that have been mapped over *d*_norm_ and the corresponding 2D fingerprint plots, respectively. Table S11 summarizes the main close contact contributions to the SSZ Hirshfeld surface area in different SSZ solids.

**Figure 16:**
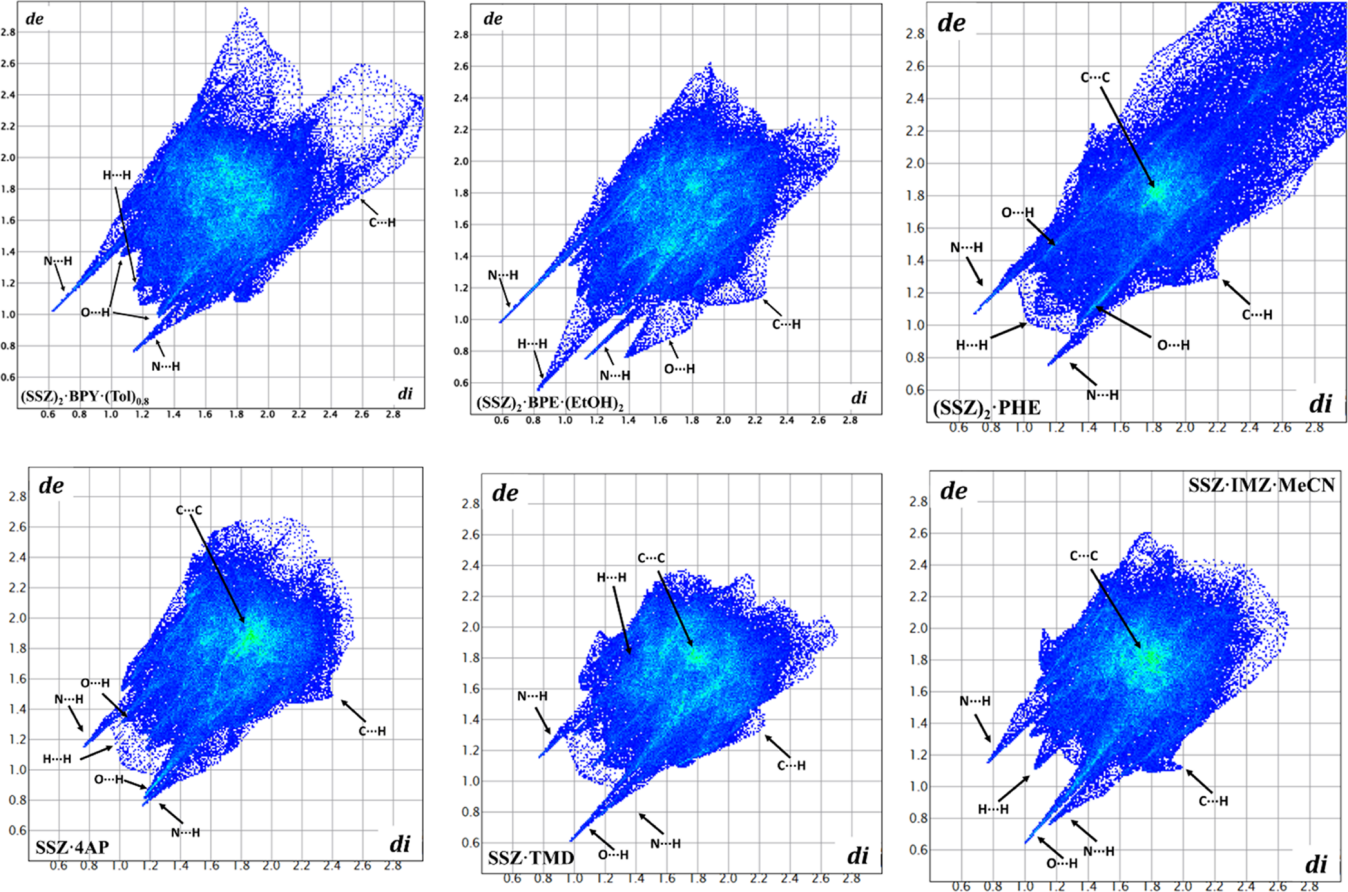
2D fingerprint plots of SSZ in six crystalline solids.

The large circular depressions (deep red) stand for the hydrogen bonding contacts (i.e., H···O and H···N), whereas other visible spots represent the H···H contacts (Figure S14).^45^ The H···H interactions have the most significant contribution to the total Hirshfeld surfaces (37.8 and 40.6%) in (SSZ)_2_·BPE·(EtOH)_2_ and (SSZ)_2_·PHE, while make up the second-largest proportion (32.8, 27.5, 32.2, and 27.1%) of the total Hirshfeld surfaces in (SSZ)_2_·BPY·(Tol)_0.8_, SSZ·4AP, SSZ·TMD, and SSZ·IMZ·MeCN. This could be because the ratios between SSZ and cocrystal/salt formers are 2:1 and 1:1, respectively; therefore, the more hydrophobic moiety (in the SSZ structure) in SSZ cocrystals leads to a higher proportion of H···H contacts compared with SSZ salts. In the 2D fingerprint plots, the longest upper spike (de > di) stands for the hydrogen bond donor, whereas the lower longest spike (de < di) represents the hydrogen bond acceptor.^35^ Therefore, when cocrystallized with BPY, BPE, and PHE, respectively, SSZ acted more as a hydrogen bond donor; when involved in salt formation, especially with TMD and IMZ, SSZ acted more as a hydrogen bond acceptor due to the proton transfer, which is in line with the crystal structure analysis.

### Highest Occupied Molecular Orbital–Lowest Unoccupied Molecular Orbital (HOMO–LUMO) Analysis

The frontier molecular orbitals play an important role in the reactivity of chemical systems, and they can also be used to predict the most reactive position in the conjugated systems.^46−48^ In particular, the energy gap between the highest occupied molecular orbital (HOMO) and the lowest unoccupied molecular orbital (LUMO) can determine the kinetic stability and chemical reactivity of the system.^48,49^

**Figure 17:**
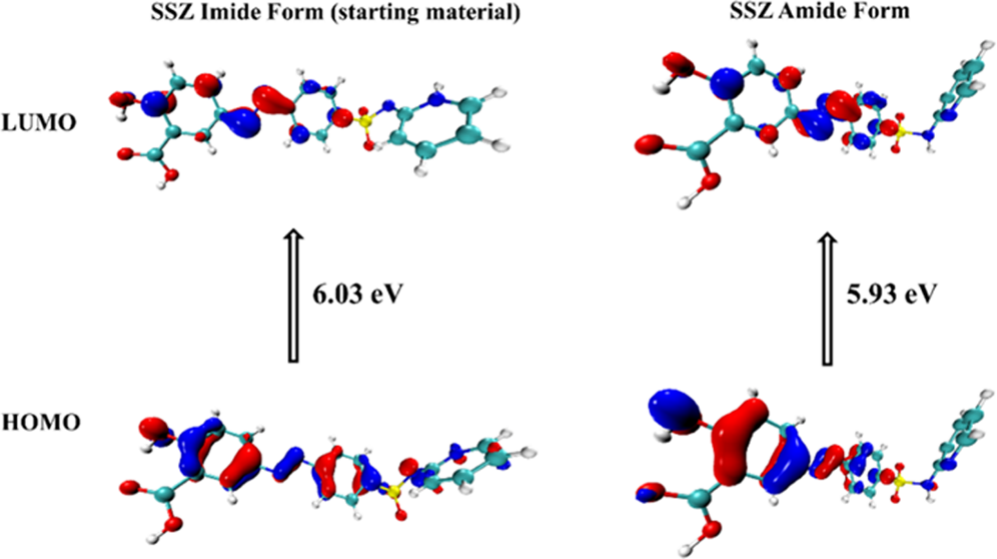
HOMOs and LUMOs of the imide (left) and amide (right) tautomers of SSZ.

**Figure 18:**
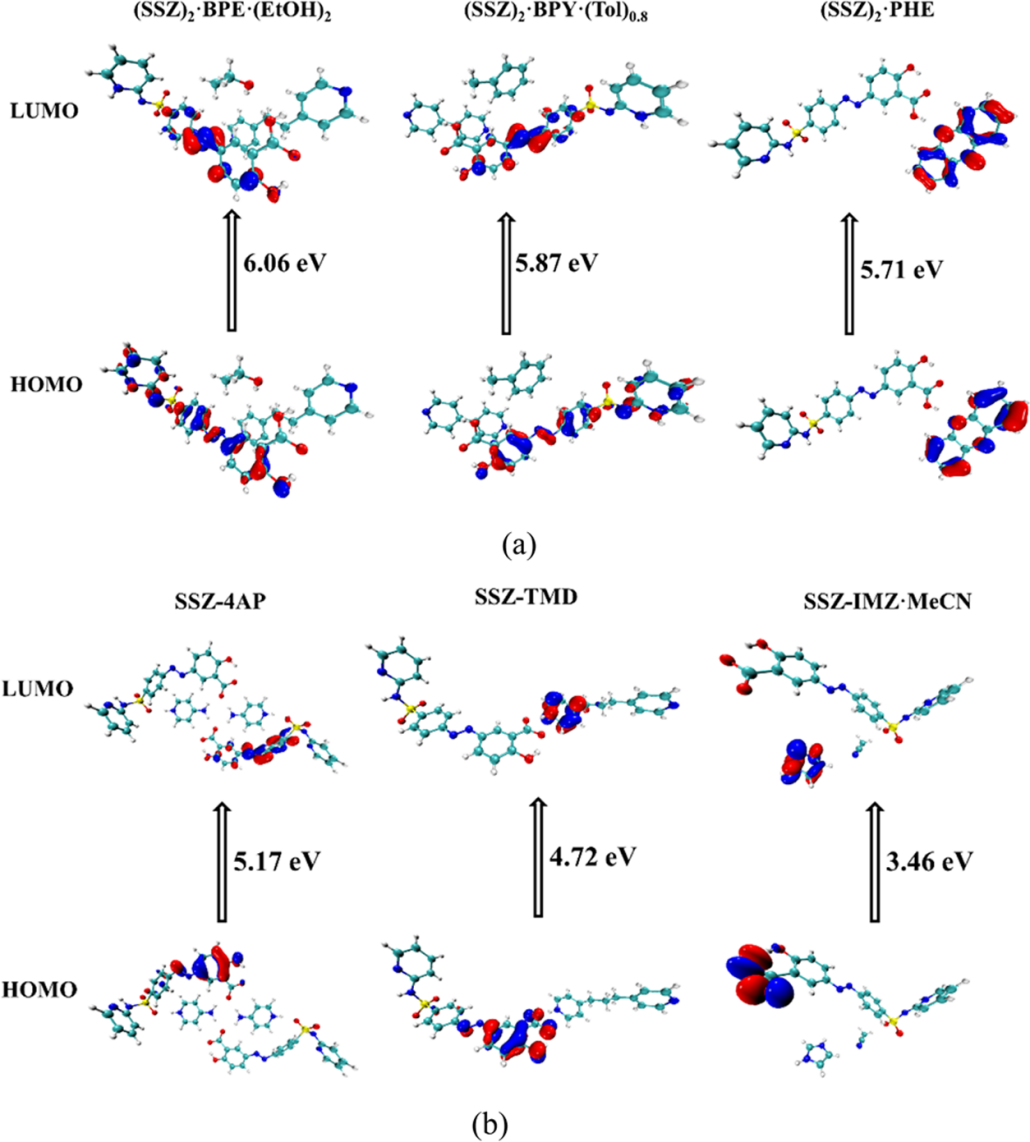
HOMOs and LUMOs of (a) the SSZ cocrystals and (b) the SSZ salts.

The distributions of the HOMO and the LUMO of the imide and amide tautomers of SSZ are similar (Figure 17). For (SSZ)_2_·BPY·(Tol)_0.8_, (SSZ)_2_·BPE·(EtOH)_2_, and SSZ·4AP, the HOMOs and the LUMOs are located on the SSZ molecule/anion (Figure 18). The HOMOs are mainly localized on the azo group and hydroxybenzoic acid moiety, whereas the LUMOs mainly spread around the benzene ring and the azo group. On the contrary, for (SSZ)_2_·PHE, both HOMO and LUMO are distributed on the PHE skeleton. For SSZ·TMD, the HOMO is mainly localized on the azo group and hydroxybenzoate moiety of SSZ^–^, and the LUMO is distributed on the protonated pyridyl ring of TMD^+^. For SSZ·IMZ·MeCN, the HOMO mainly spreads around the carboxylate group of the SSZ^–^ anion and the LUMO is located on the imidazolium moiety.

It has been reported that larger HOMO and LUMO energy gaps lead to higher chemical stability and lower chemical reactivity, and vice versa.^48^ As illustrated in Figure 17, the energy gap in the imide is greater than the amide for pure SSZ, suggesting that the imide form is more stable than the amide form. This may be supported by the fact that the amide form was the first synthesized polymorph at ambient environment, while the imide form was obtained at high temperature and high pressure. The order of energy gaps in the SSZ cocrystals and salts is (SSZ)_2_·BPE·(EtOH)_2_ > (SSZ)_2_·BPY·(Tol)_0.8_ > (SSZ)_2_·PHE > SSZ·4AP > SSZ·TMD > SSZ·IMZ·MeCN. Notably, the cocrystals are more stable than the salts, and there is a significant difference for the solvated salt SSZ·IMZ·MeCN.

## Conclusions

Sulfasalazine has a strong tendency to form multicomponent crystalline materials with a variety of cocrystal/salt formers because it has multiple hydrogen bond acceptor and donor sites in the structure and exhibits tautomerism. In these multicomponent forms, SSZ exists in different conformations. The *R*_2_^2^(7):*R*_2_^2^(8):*R*_2_^2^(7) motif between two adjacent SSZ occurs in all the multicomponent forms, presenting an ADAD array in three unsolvated SSZ solids, while the AADD array is observed in solvated cocrystals and salt (SSZ)_2_·BPY·(Tol)_0.8_, (SSZ)_2_·BPE·(EtOH)_2_, and SSZ·IMZ·MeCN, which is also present in the imide form of SSZ. In addition, the azo group from SSZ participates in hydrogen bonding only in SSZ-TMD. Hirshfeld surface analysis indicates that SSZ acts as a hydrogen bond donor when forming cocrystals and as a hydrogen bond acceptor when it forms salts, which is consistent with the occurence of proton transfer determined by SCXRD results. HOMO–LUMO results suggested the cocrystals are chemically more stable than the salts.

## Supporting Information Available

DSC traces, TGA plots, IR spectra, PXRD patterns, ellipsoid plots, diagrams of π–π interactions of pure SSZ and multicomponent crystals, 3D *d*_norm_ surfaces, a summary of experiments for SSZ cocrystals and salts, table of crystallographic parameters of pure SSZ and multicomponent forms, selected geometric parameters of pure SSZ and six cocrystals and salts, and summary of the various contact contributions to the SSZ Hirshfeld surface area in SSZ cocrystals and salts (PDF)
