# Supplementary material for: Exploring the Crystal Structure Landscape of Sulfasalazine through Various Multicomponent Crystals
Source: Cryst Growth Des. 2023 Jul 19;23(8):5446–61. doi: 10.1021/acs.cgd.2c01403 (PMC10401639; doi:10.1021/acs.cgd.2c01403)
Supplement: Supplementary file 1 — cg2c01403_si_001.pdf [file cg2c01403_si_001.pdf]

Supporting Information

Exploring the Crystal Structure Landscape of Sulfasalazine through Various Multicomponent Crystals

Shan Huang, [†,‡] *Vinay K. R. Cheemarla,* [†] *Davide Tiana,* [†] *and Simon E. Lawrence*[†,‡,*]

[†]School of Chemistry, Synthesis and Solid State Pharmaceutical Centre, University College Cork, Cork T12 K8AF, Ireland

[‡]Analytical and Biological Chemistry Research Facility, University College Cork, Cork T12 K8AF, Ireland

Contents

Characterizations of SSZ cocrystals and salts	2
Ellipsoid plots of SSZ cocrystals and salts	12
π-π interactions of SSZ polymorphs, cocrystals and salts	14
3D d_{norm} surfaces of SSZ in six crystalline solids	18
Summary of successful experiments for SSZ cocrystals and salts	19
Hydrogen bond and π–π interaction geometries in SSZ crystals	20
Selected geometric parameters in SSZ crystals	29
Summary of the various contact contributions to the SSZ Hirshfeld surface area in different cocrystals and salts	30
References	31

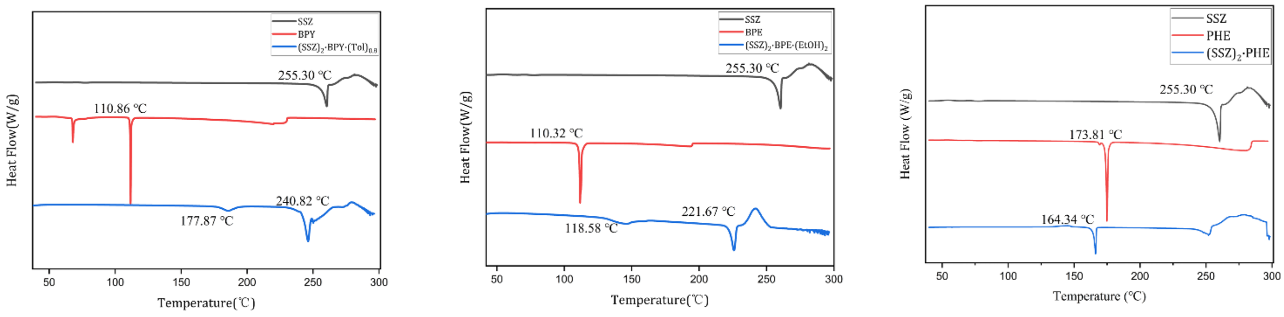

(a)

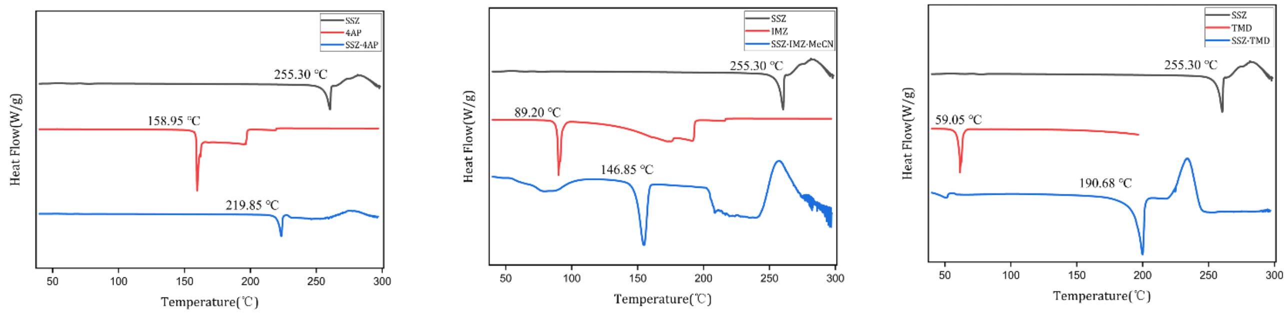

(b)

Figure S1. DSC traces of (a) SSZ, BPY, BPE, PHE and $(SSZ)_2 \cdot BPY \cdot (Tol)_{0.8}$, $(SSZ)_2 \cdot BPE \cdot (EtOH)_2$ and $(SSZ)_2 \cdot PHE$, and (b) SSZ, 4AP, TMD, IMZ and SSZ·4AP, SSZ·TMD and SSZ·IMZ·MeCN.

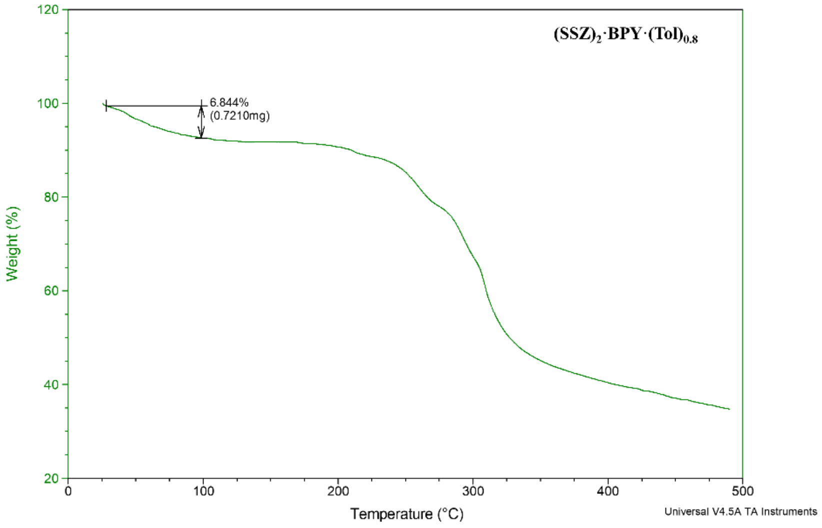
(SSZ)2·BPY·(Tol)0.8
6.844%
(0.7210mg)
Weight (%)
Temperature (°C)
Universal V4.5A TA Instruments
120
100
80
60
40
20
0
100
200
300
400
500

(a)

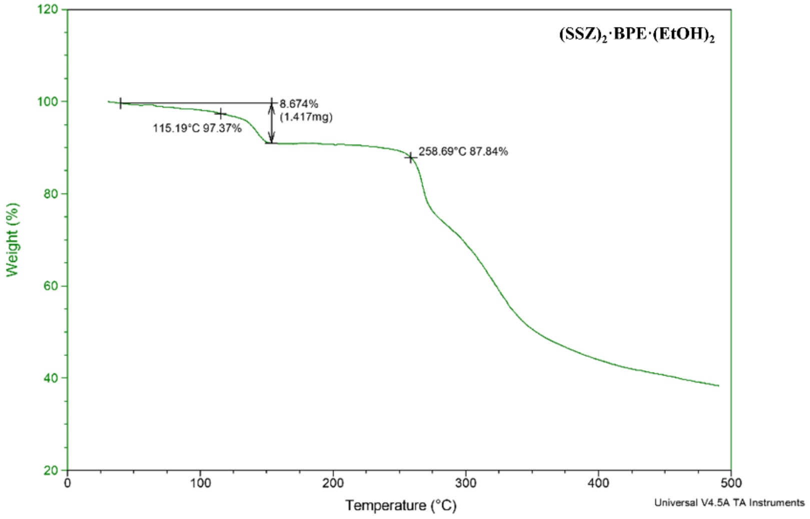
(SSZ)2·BPE·(EtOH)2
8.674%
(1.417mg)
115.19°C 97.37%
258.69°C 87.84%
Weight (%)
Temperature (°C)
Universal V4.5A TA Instruments
120
100
80
60
40
20
0
100
200
300
400
500

(b)

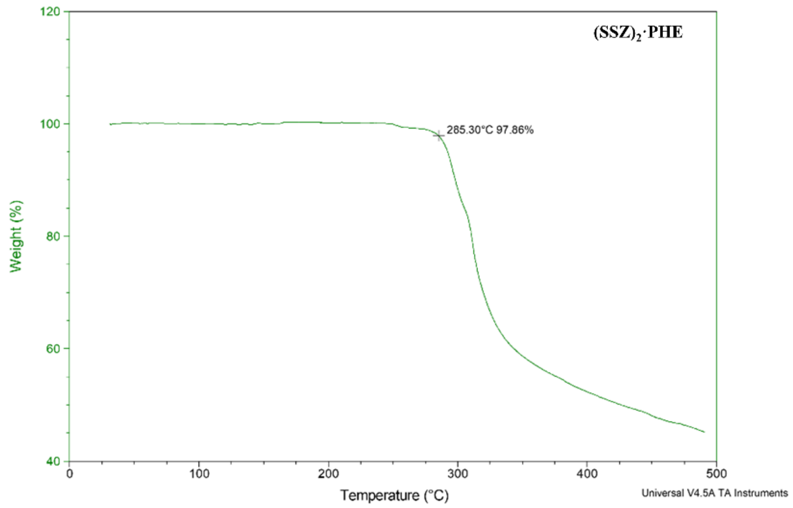
(SSZ)2·PHE
285.30°C 97.86%
Weight (%)
Temperature (°C)
Universal V4.5A TA Instruments

(c)

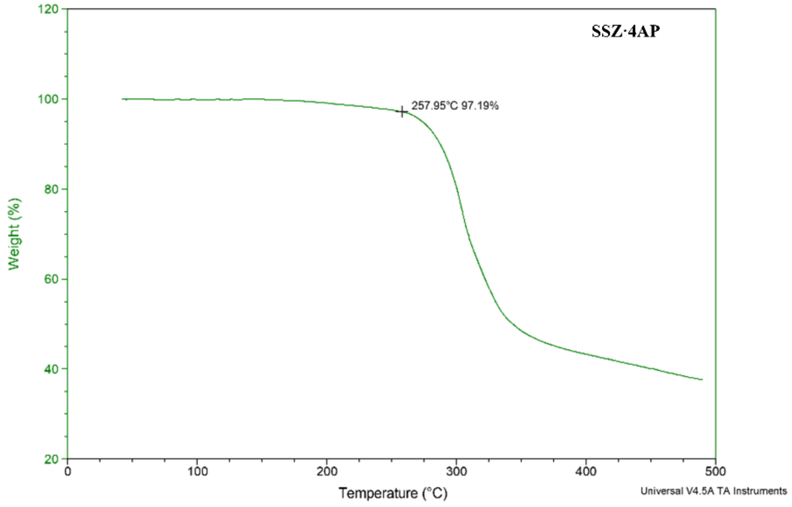
SSZ·4AP
257.95°C 97.19%
Weight (%)
Temperature (°C)
Universal V4.5A TA Instruments

(d)

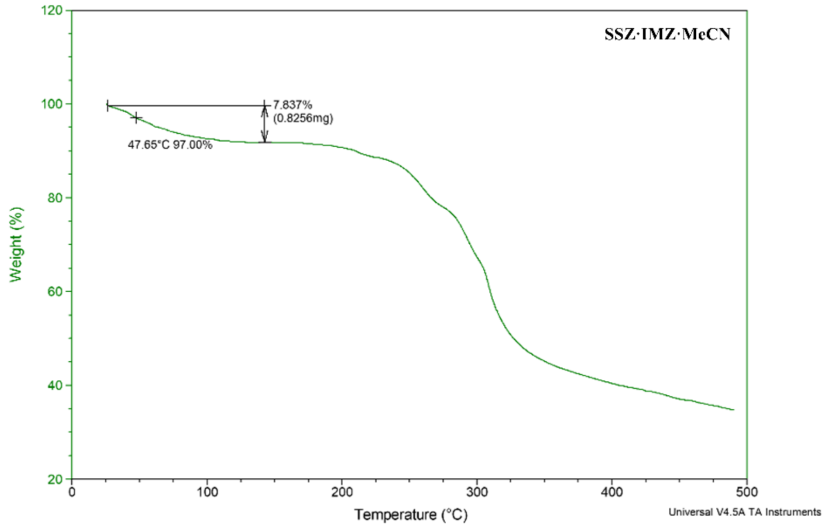

(e)

Figure S2. TGA plots of (a) $(SSZ)_2$·BPY·$(Tol)_{0.8}$, (b) $(SSZ)_2$·BPE·$(EtOH)_2$ cocrystal solvate, (c) $(SSZ)_2$·PHE cocrystal, (d) SSZ·4AP salt, and (e) SSZ·IMZ·MeCN salt solvate.

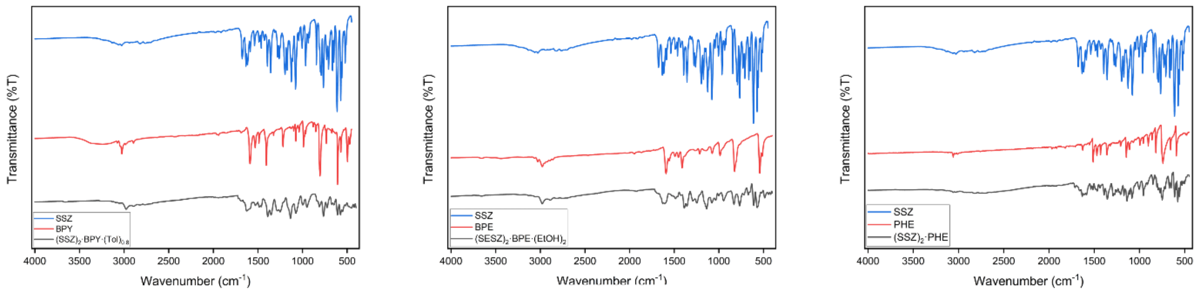

(a)

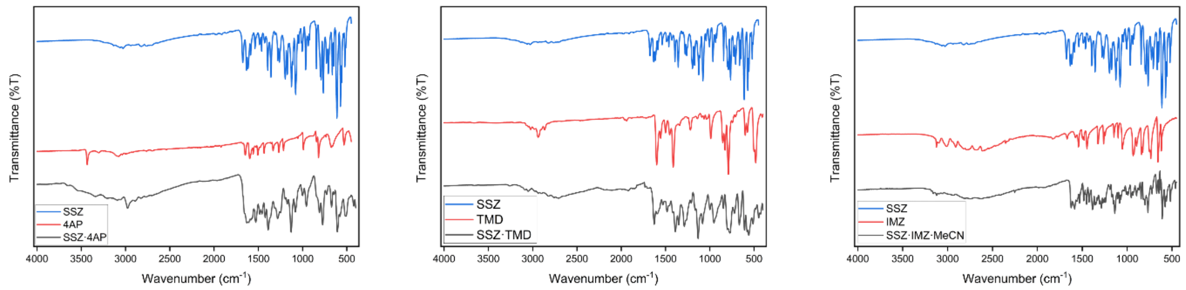

(b)

Figure S3. IR spectra of (a) SSZ, BPY, BPE, PHE and $(SSZ)_2$·BPY·$(Tol)_{0.8}$, $(SSZ)_2$·BPE·$(EtOH)_2$ and $(SSZ)_2$·PHE, and (b) SSZ, 4AP, TMD, IMZ and SSZ·4AP, SSZ·TMD and SSZ·IMZ·MeCN.

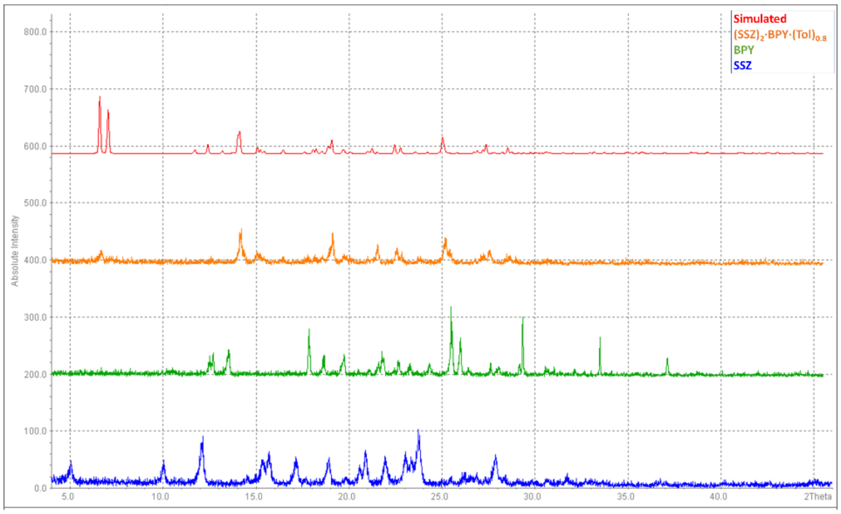
Simulated
(SSZ)2·BPY·(Tol)0.8
BPY
SSZ
Absolute Intensity
800.0
700.0
600.0
500.0
400.0
300.0
200.0
100.0
0.0
5.0
10.0
15.0
20.0
25.0
30.0
35.0
40.0
2Theta

(a)

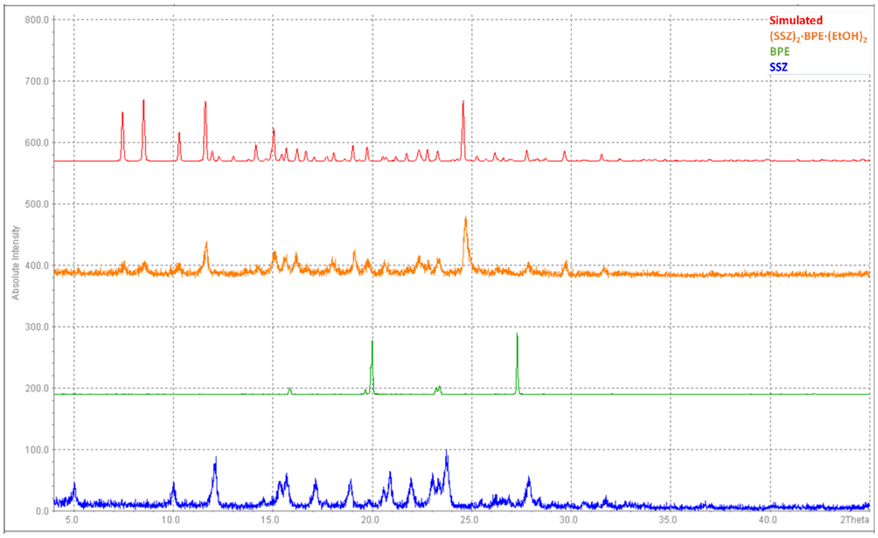
Simulated
(SSZ)2·BPE·(EtOH)2
BPE
SSZ
Absolute Intensity
800.0
700.0
600.0
500.0
400.0
300.0
200.0
100.0
0.0
5.0
10.0
15.0
20.0
25.0
30.0
35.0
40.0
2Theta

(b)

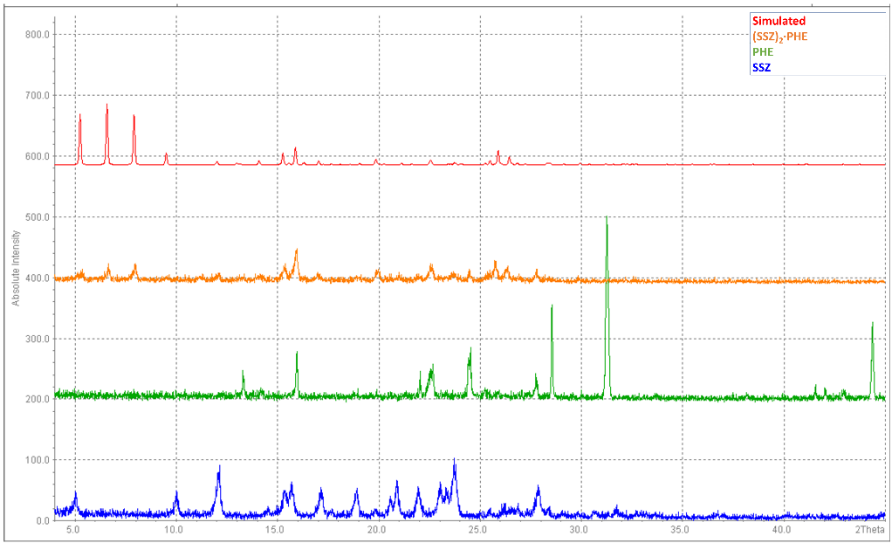
Simulated
(SSZ)$_2$·PHE
PHE
SSZ
Absolute Intensity
800.0
700.0
600.0
500.0
400.0
300.0
200.0
100.0
0.0
5.0
10.0
15.0
20.0
25.0
30.0
35.0
40.0
2Theta

(c)

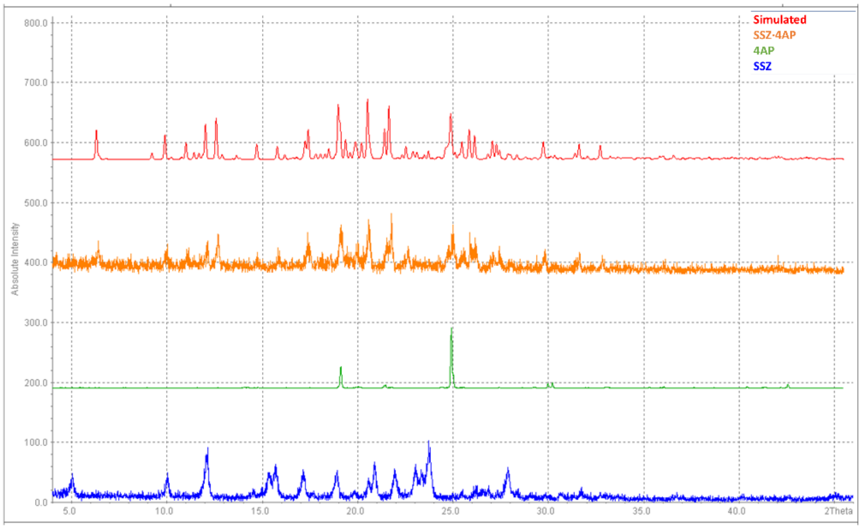
Simulated
SSZ·4AP
4AP
SSZ
Absolute Intensity
800.0
700.0
600.0
500.0
400.0
300.0
200.0
100.0
0.0
5.0
10.0
15.0
20.0
25.0
30.0
35.0
40.0
2Theta

(d)

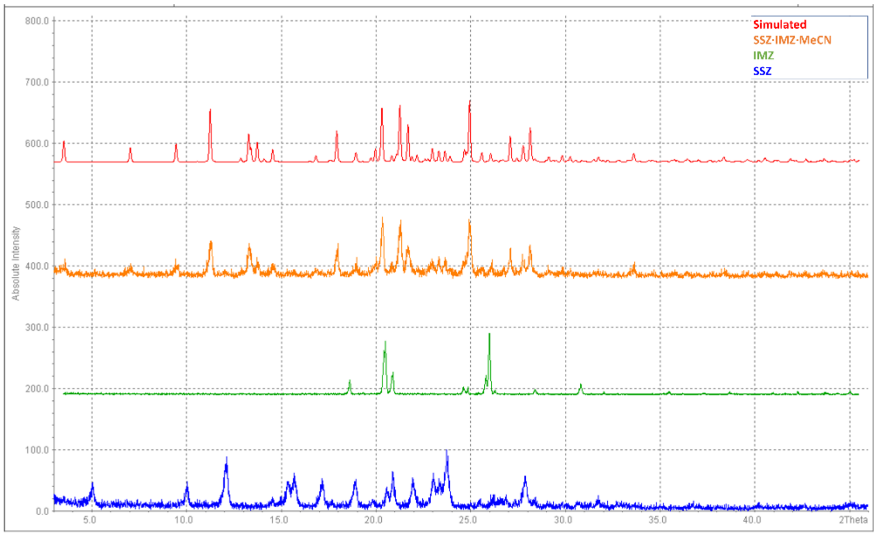

(e)

Figure S4. PXRD patterns of (a) $(SSZ)_2 \cdot BPY \cdot (Tol)_{0.8}$, (b) $(SSZ)_2 \cdot BPE \cdot (EtOH)_2$, (c) $(SSZ)_2 \cdot PHE$, (d) SSZ·4AP, and (e) SSZ·IMZ·MeCN.

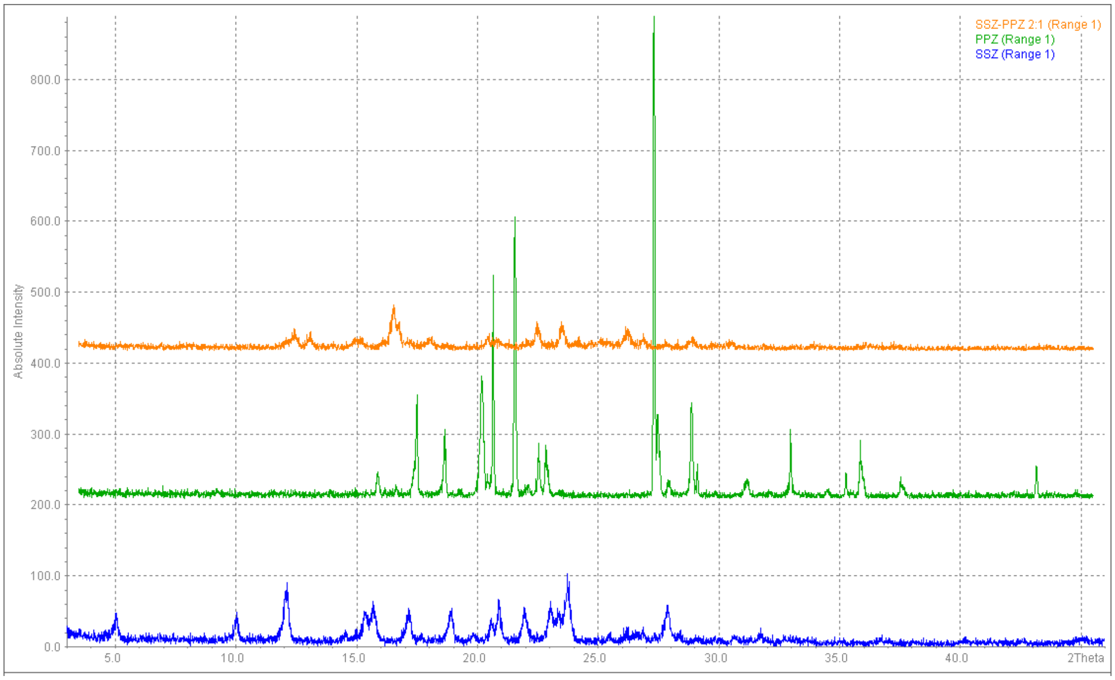
SSZ-PPZ 2:1 (Range 1)
PPZ (Range 1)
SSZ (Range 1)
Absolute Intensity
2Theta

(a)

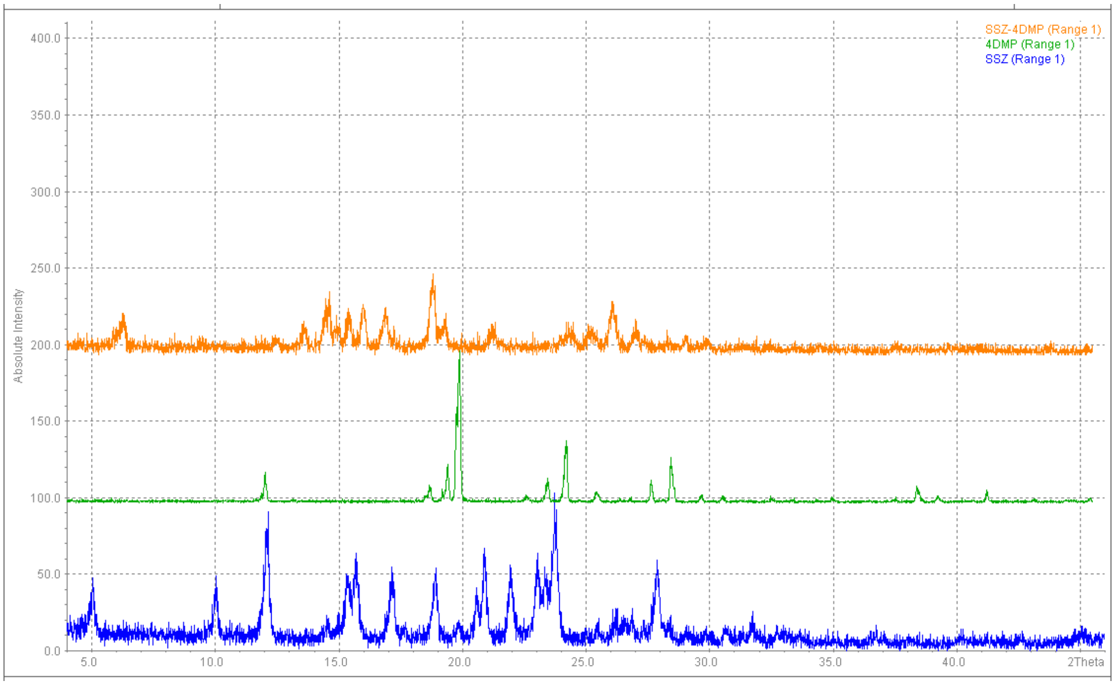
SSZ-4DMP (Range 1)
4DMP (Range 1)
SSZ (Range 1)
Absolute Intensity
2Theta

(b)

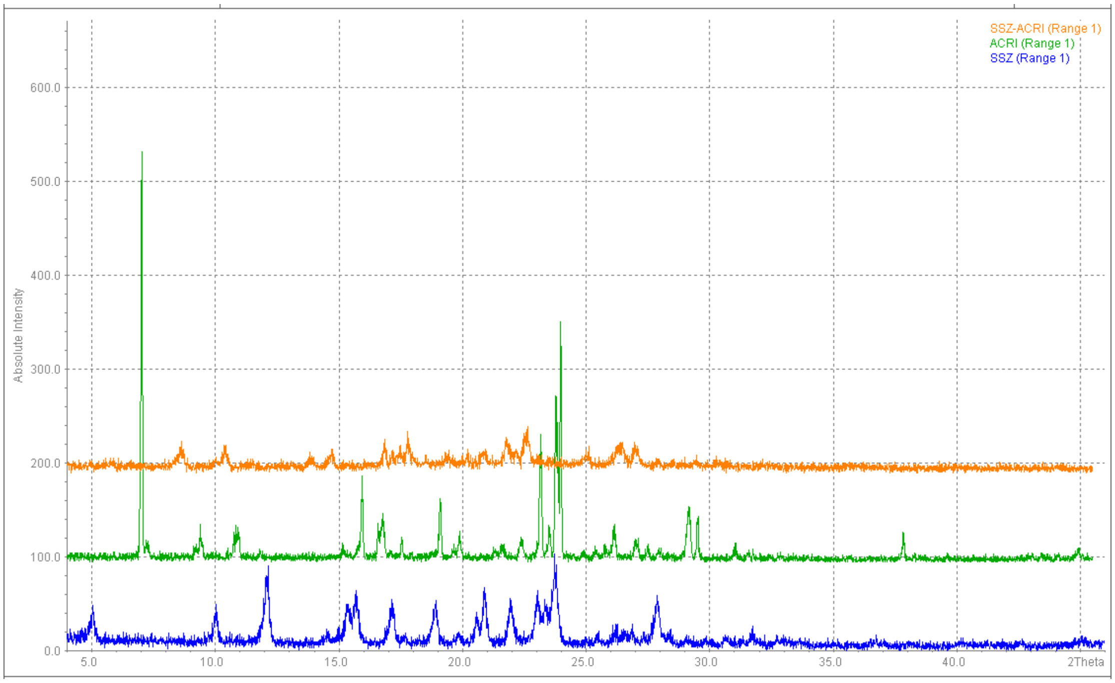

(c)

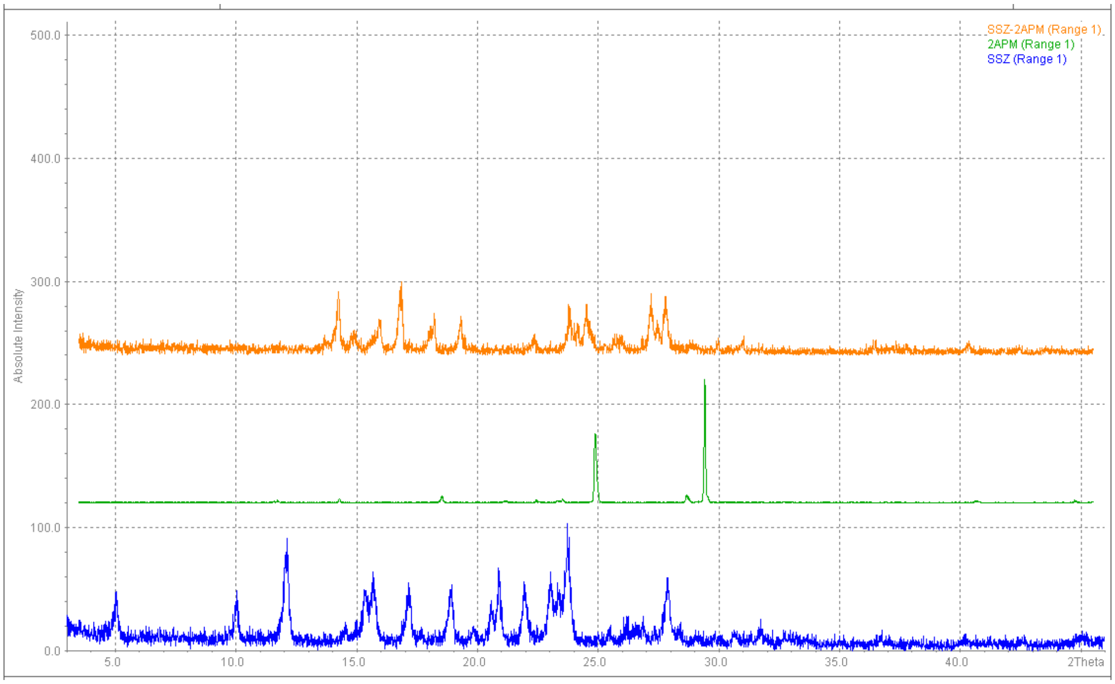

(d)

Figure S5. PXRD patterns of (a) SSZ-PPZ, (b) SSZ-4DMP, (c) SSZ-ACRI and (d) SSZ-2AMP.

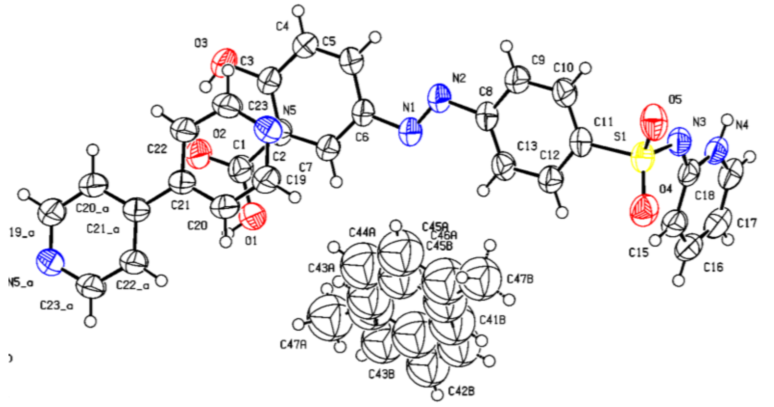
O3
C3
C4
C5
N1
N2
C8
C9
C10
O5
N3
N4
C23
N5
C6
C11
S1
C22
O2
C1
C2
C7
C13
C12
C20_a
C21_a
C21
C19
O4
C18
19_a
C20
O1
C17
N5_a
C22_a
C15
C16
C23_a
C44A
C45A
C46A
C45B
C43A
C47B
C41B
C47A
C43B
C42B

(a)

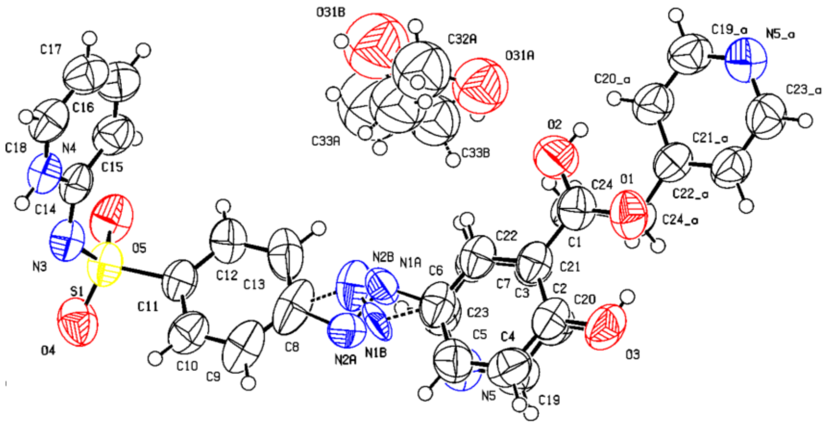
O31B
C32A
O31A
C19_a
N5_a
C17
C20_a
C23_a
C16
C33A
C33B
O2
C18
N4
C15
C21_a
C14
C24
O1
C22_a
C24_a
O5
C22
N3
C12
N2B
N1A
C1
S1
C13
C6
C21
C11
C7
C3
C2
C20
C23
O4
C10
C9
C8
N2A
N1B
C5
C4
O3
N5
C19

(b)

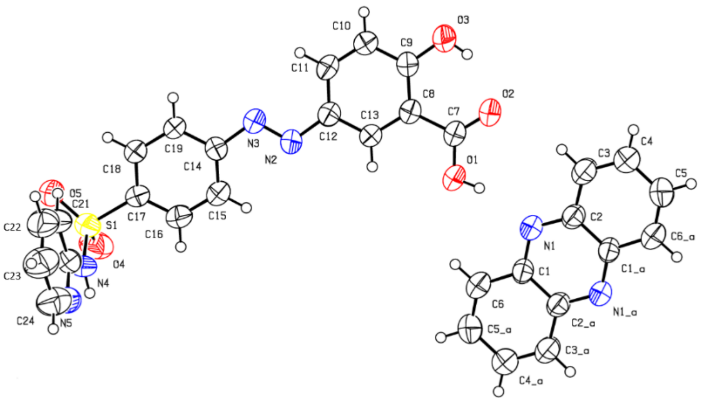
C10
O3
C9
C11
C8
O2
C13
C7
N3
C12
C19
C14
N2
O1
C3
C4
C18
C5
O5
C21
C17
C15
C2
C22
S1
C16
N1
C6_a
O4
N4
C1
C1_a
C23
C6
N1_a
C24
N5
C5_a
C2_a
C3_a
C4_a

(c)

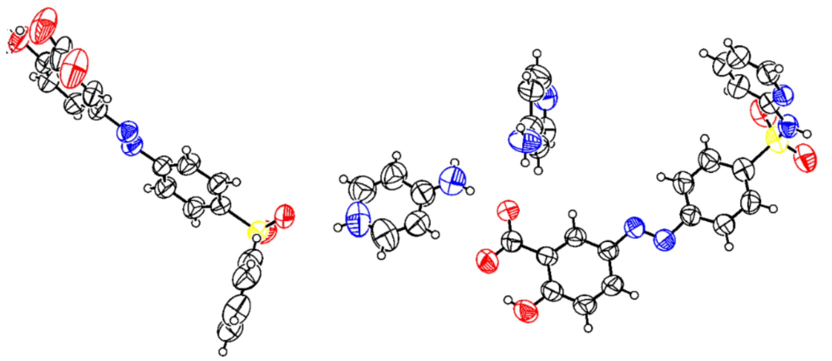

(d)

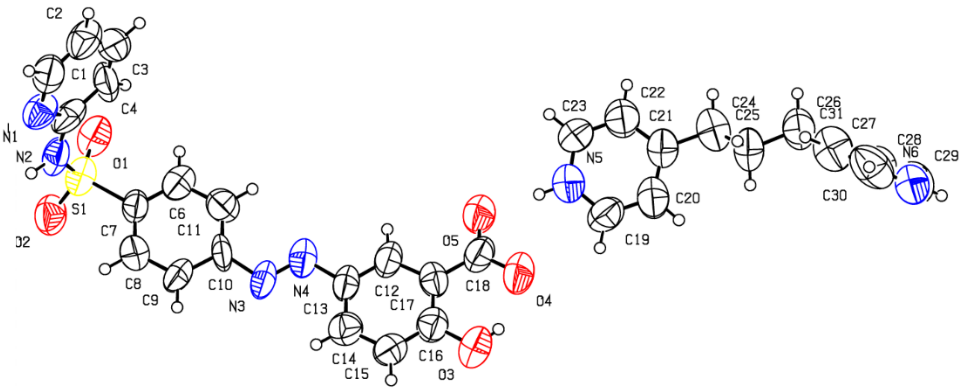

(e)

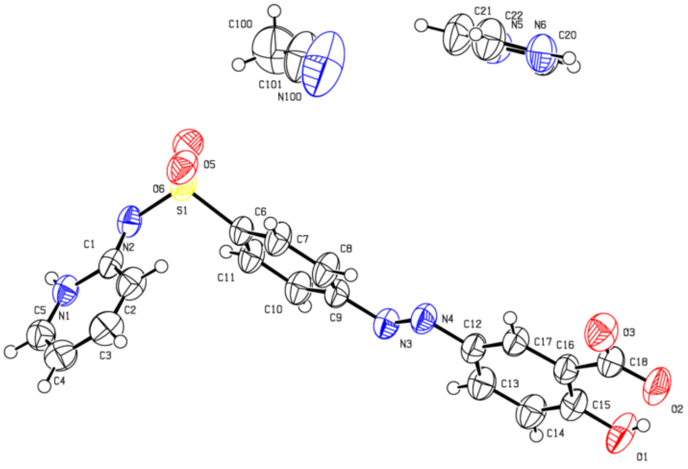

(f)

Figure S6. Ellipsoid plots of (a) $(SSZ)_2$·BPY·$(Tol)_{0.8}$, (b) $(SSZ)_2$·BPE·$(EtOH)_2$, (c) $(SSZ)_2$·PHE, (d) SSZ·4AP, (e) SSZ·TMD and (f) SSZ·IMZ·MeCN.

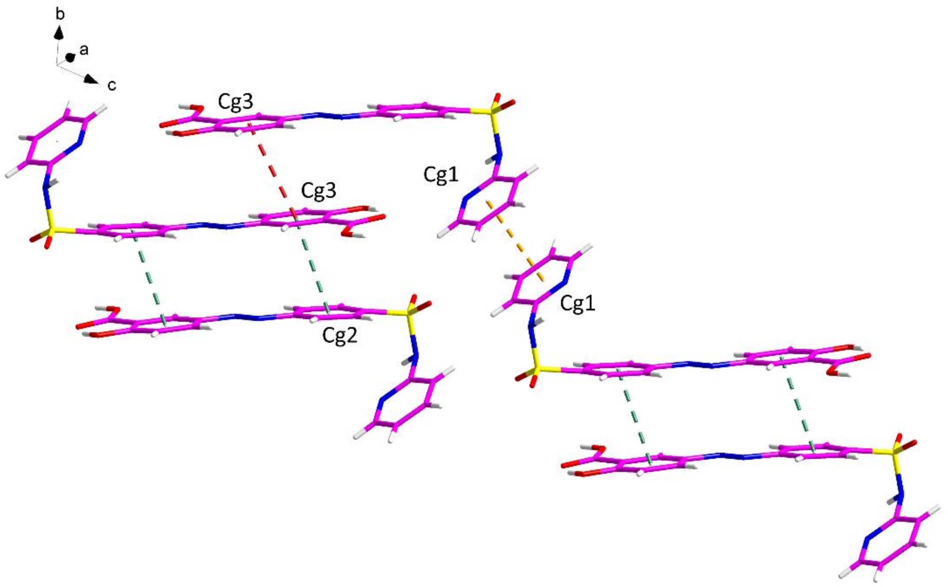

(a)

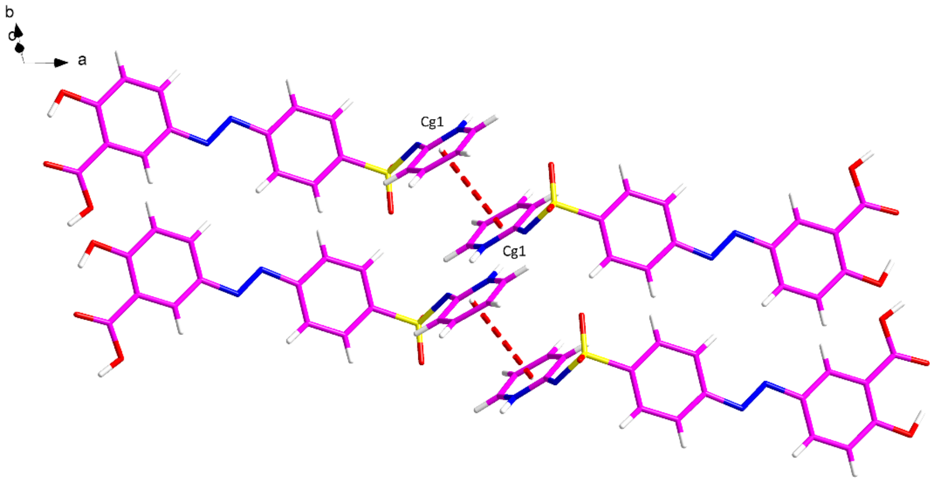

(b)

Figure S7. π-π interactions of (a) in the SSZ triclinic amide form, and (b) in the SSZ monoclinic imide form as indicated by dashed lines (hydrogen bonding is not displayed for clarity).

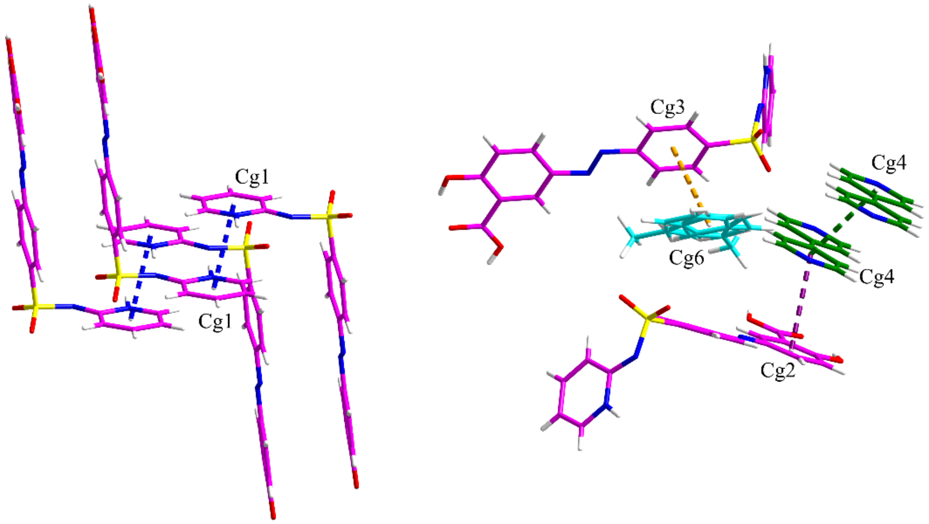

Figure S8. π-π interactions of $(SSZ)_2$·BPY·$(Tol)_{0.8}$ cocrystal solvate.

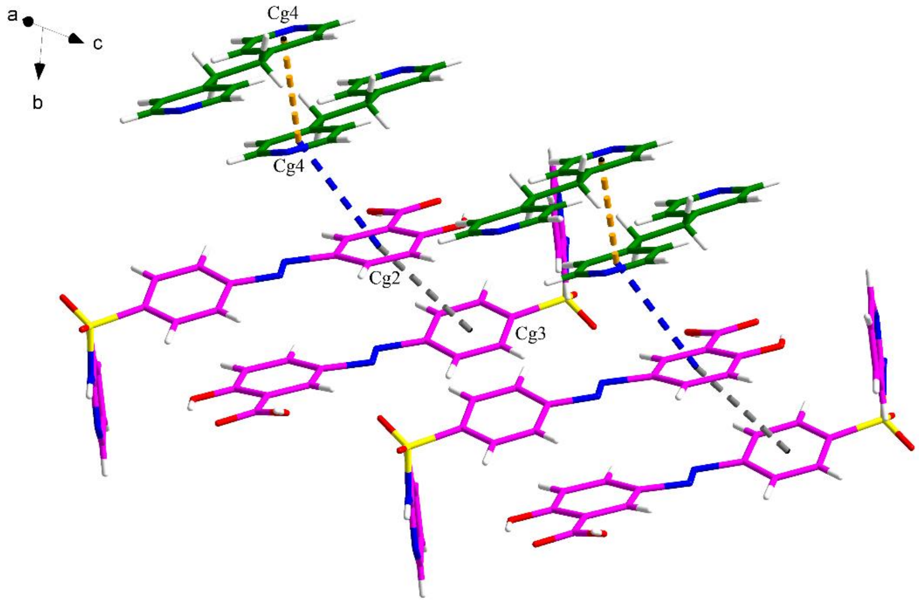

Figure S9. π-π interactions of $(SSZ)_2$·BPE·$(EtOH)_2$ cocrystal solvate.

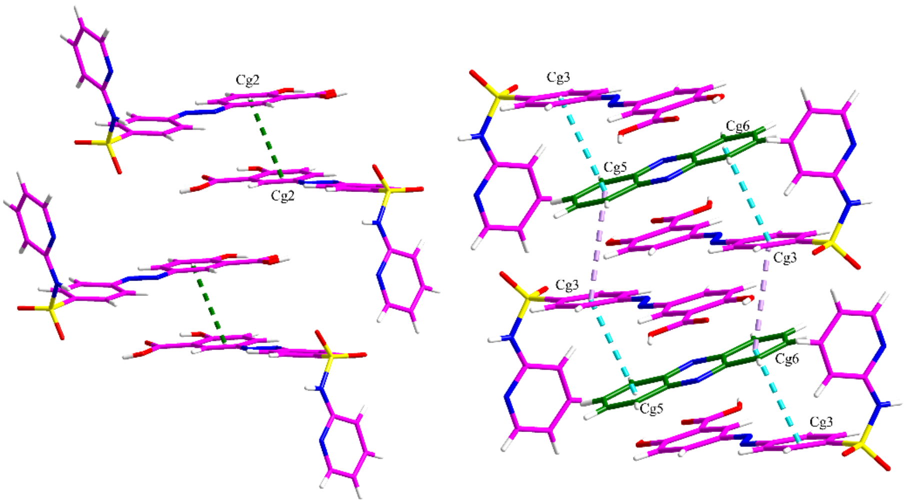

Figure S10. π-π interactions of $(SSZ)_2$·PHE cocrystal.

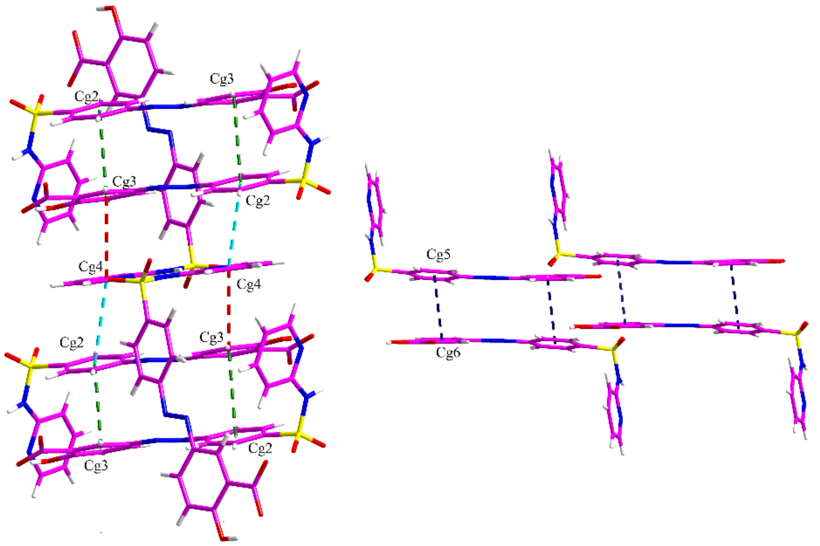

Figure S11. π-π interactions of SSZ·4AP salt.

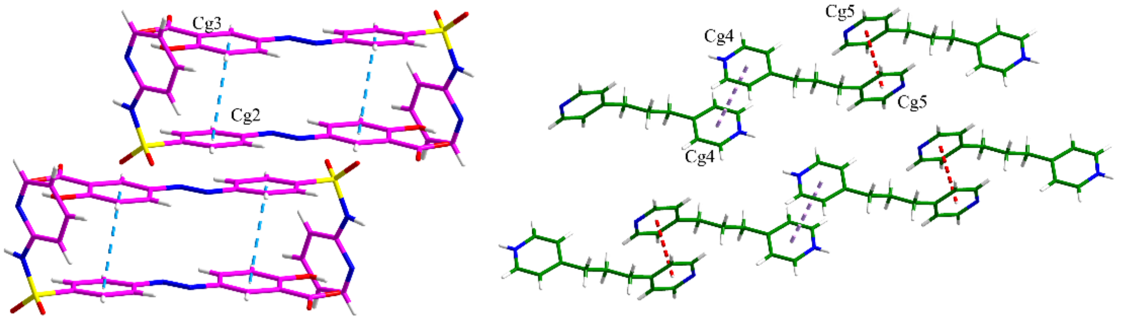

Figure S12. π-π interactions of SSZ·TMD salt.

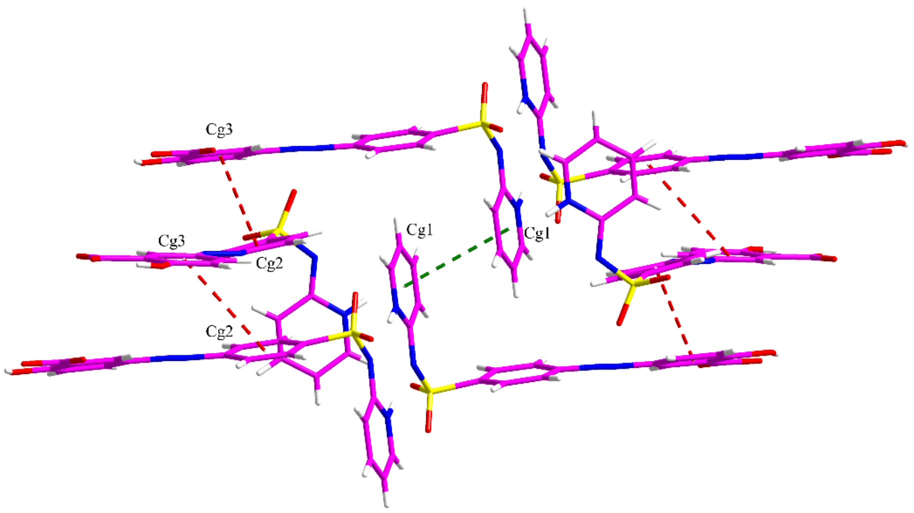

Figure S13. π-π interactions of SSZ·IMZ·MeCN salt solvate.

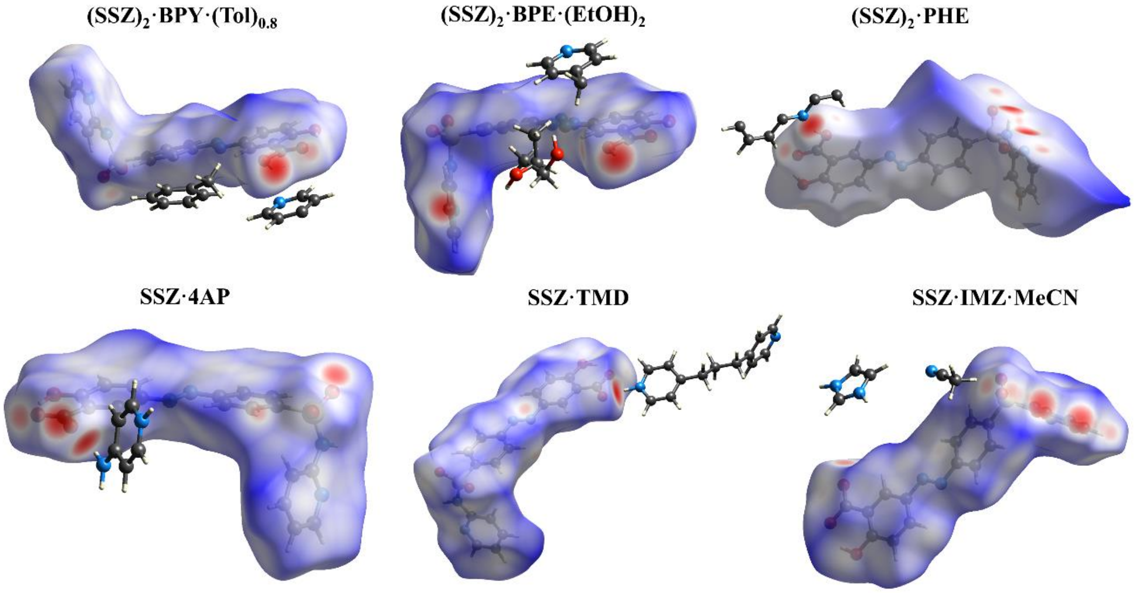

Figure S14. 3D d_{norm} surfaces of SSZ in six crystalline solids.

Table S1. Summary of successful experiments for SSZ cocrystals and salts

**Crystal forms**	**SSZ**	**CCF/Salt former**	**Method**	**Solvent**
$(SSZ)_2$·BPY	398.4 mg, 1 mmol	78.1 mg, 0.5 mmol	Slurry	MeOH 3 mL
$(SSZ)_2$·BPE·$(EtOH)_2$	398.4 mg, 1 mmol	92.1 mg, 0.5 mmol	Slurry	EtOH 3 mL
$(SSZ)_2$·PHE	398.4 mg, 1 mmol	90.1 mg, 0.5 mmol	Slurry	EtOH 3 mL
SSZ·4AP	398.4 mg, 1 mmol	94.1 mg, 1 mmol	Slurry	MeOH 3 mL
SSZ·IMZ	398.4 mg, 1 mmol	68.1 mg, 1mmol	LAG	MeCN 30 μL
SSZ-PPZ	398.4 mg, 1 mmol	43.1 mg, 0.5 mmol	Slurry	MeOH 3 mL
SSZ-2APM	398.4 mg, 1 mmol	95.1 mg, 1 mmol	Slurry	MeOH 3 mL
SSZ-ACRI	398.4 mg, 1 mmol	179.1 mg, 1 mmol	Slurry	MeOH 3 mL
SSZ-4DMP	398.4 mg, 1 mmol	94.1 mg, 1 mmol	Slurry	MeOH 3 mL

Table S2. Hydrogen bond and π–π interaction geometries (Å, °) in the SSZ triclinic amide form[1,a]

	Type	D-H···A	D-H	H···A	D···A	D-H···A	ARU (J)
1		N7-H7···O27	0.993(17)	1.966(16)	2.948(2)	169.3(17)	[2655.01]
2		O26-H26···N1	1.01(2)	1.62(2)	2.623(2)	173(2)	[2655.01]
3	Intra	O28-H28···O27	0.91(3)	1.76(3)	2.604(2)	154(3)	
4		C4-H4···O10	0.93	2.6	3.416(3)	147	[2666.01]
5		C6-H6···O9	0.93	2.58	3.403(3)	147	[1445.01]
6	Intra	C12-H12···O10	0.93	2.6	2.950(2)	103	
7		C13-H13···O9	0.93	2.47	3.190(3)	135	[1455.01]
**Cg(I)**	**Cg(J)**	**Cg-Cg**	**Interplanar distance**		**Dihedral Angle**	**Beta**	**ARU (J)**
1	1	3.6802(13)	3.4095(8)		0.03(10)	22.1	[2656.01]
2	3	3.8848(12)	3.4943(8)		3.87(9)	27.1	[2665.01]
3	3	4.0449(12)	3.5925(7)		0.00(9)	27.4	[2555.01]

[a] Symmetry codes: [2666.] = 1-x, 1-y, 1-z; [2655.] = 1-x, -y, -z; [1455.] = -1+x, y, z; [1445.] = -1+x, -1+y, z; [2656] = 1-x, -y, 1-z; [2665] = 1-x, 1-y, -z; [2555] = -x, -y, -z. Cg1, Cg2 and Cg3 are centroids of N1, C2–C6, C11–C16 and C19–C24, respectively. Cg(I) = plane number I; Cg–Cg = distance between ring centroids (Ang.). Beta is the displacement angle between the ring normal of plane I and the centroid vector.

Table S3. Hydrogen bond and π–π interaction geometries (Å, °) in the SSZ monoclinic imide form[2,a]

	Type	D-H···A	D-H	H···A	D···A	D-H···A	ARU (J)
1		N1-H1N···N2	0.90(3)	1.98(3)	2.878(4)	173(3)	[3766.01]
2	Intra	O3-H3O···O4	0.91(4)	1.75(3)	2.612(4)	157(4)	
3		O5-H5O···O2	0.91(4)	1.77(4)	2.635(4)	158(4)	[2645.01]
4		C1-H1···O1	0.93	2.56	2.945(5)	105	[3756.01]
5		C1-H1···O2	0.93	2.54	3.161(4)	124	[3766.01]
6		C3-H3···O4	0.93	2.48	3.404(5)	171	[3656.01]
7	Intra	C4-H4···O1	0.93	2.55	2.982(5)	109	
8	Intra	C7-H7···O1	0.93	2.49	2.869(5)	105	
9	Intra	C18-H18···O5	0.93	2.38	2.695(5)	100	

Cg(I)	Cg(J)	Cg-Cg	Interplanar distance	Dihedral Angle	Beta	ARU (J)
1	1	4.267(2)	3.9237(15)	0.00(18)	23.1	[3756.01]

[a] Symmetry codes: [3766.] = 2-x, 1-y, 1-z; [2645.] = 1-x, -1/2+y, 1/2-z; [3756.] = 2-x, -y, 1-z; [3656.] = 1-x, -y, 1-z; [3756] = 2-x, -y, 1-z. Cg1 is centroid of N1, C1–C5. Cg(I) = plane number I; Cg–Cg = distance between ring centroids (Ang.). Beta is the displacement angle between the ring normal of plane I and the centroid vector.

Table S4. Hydrogen bond and π–π interaction geometries (Å, °) in the $(SSZ)_2{\cdot}BPY{\cdot}(Tol)_{0.8}$ cocrystal solvate [a]

	Type	D-H···A	D-H	H···A	D···A	D-H···A	ARU (J)
1		O1-H1···N5	0.82	1.79	2.613(2)	176	[1545.02]
2	Intra	O3-H3A···O2	0.82	1.79	2.514(2)	147	
3		N4-H4N···N3	0.862(14)	2.074(14)	2.927(2)	169.9(19)	[3686.01]
4	Intra	C10-H10···O5	0.93	2.59	2.933(3)	102	
5	Intra	C15-H15···O4	0.93	2.32	2.928(3)	122	
6		C18-H18···O5	0.93	2.55	3.224(3)	130	[3686.01]
7		C20-H20···O4	0.93	2.45	3.338(3)	160	[2545.01]
8		C23-H23···O2	0.93	2.4	3.082(3)	130	[1565.01]

Cg(I)	Cg(J)	Cg-Cg	Interplanar distance	Dihedral Angle	Beta	ARU (J)
1	1	3.4630(11)	3.2495 (8)	0.02(9)	20.2	[3676.01]
2	4	4.0140(12)	3.4478(8)	4.43(10)	27.6	[1555.02]
3	6	4.019(7)	3.6470(8)	23.6(6)	15.2	[1565.04]
4	4	3.8910(13)	3.5740(10)	0.00(11)	23.3	[3675.02]

[a] Symmetry codes: [3686.] = [3_686] = 1-x, 3-y, 1-z; [1545.] = [1_545] = x, -1+y, z; [1565.] = [1_565] = x, 1+y, z; [2545.] = [2_545] = 1/2-x, -1+y, 1/2-z; [3676] = 1-x, 2-y, 1-z; [1555.] = x, y, z; [1565] = x, 1+y, z; [3675] = 1-x, 2-y, -z. Cg1, Cg2, C3, C4 and Cg6 are centroids of N4/C14–C18, C2–C7, C8–C13, N5/C19–C23 and C41B–C46B, respectively. Cg(I) = plane number I; Cg–Cg = distance between ring centroids (Ang.). Beta is the displacement angle between the ring normal of plane I and the centroid vector.

Table S5. Hydrogen bond and π–π interaction geometries (Å, °) in the $(SSZ)_2$·BPE·$(EtOH)_2$ cocrystal solvate [a]

	Type	D-H···A	D-H	H···A	D···A	D-H···A	ARU (J)
1		O2-H2···N5	0.82	1.74	2.556(4)	171	[1655.02]
2	Intra	O3-H3···O1	0.82	1.83	2.551(4)	146	
3		N4-H4N···N3	0.86(3)	2.04(3)	2.891(4)	172(3)	[2557.01]
4	Intra	C7-H7···O2	0.93	2.44	2.762(4)	100	
5	Intra	C12-H12···O5	0.93	2.59	2.935(4)	103	
6	Intra	C15-H15···O5	0.93	2.42	3.009(5)	121	
7		C16-H16···O31A	0.93	2.44	3.194(7)	138	[2667.03]
8		C18-H18···O4	0.93	2.47	3.174(5)	132	[2557.01]
9		C22-H22···O5	0.93	2.51	3.310(4)	144	[2567.01]

Cg(I)	Cg(J)	Cg-Cg	Interplanar distance	Dihedral Angle	Beta	ARU (J)
2	3	4.290(2)	3.6647(14)	15.42(17)	36.1	[2666.01]
2	4	4.140(2)	3.8293(14)	12.32(17)	28.9	[1555.02]
4	4	3.943(2)	3.5232(15)	0.00(18)	26.7	[2576.02]

[a] Symmetry codes: [1555] = x, y, z; [1655.] = [1_655] = 1+x, y, z; [2557.] = [2_557] = -x, -y, 2-z; [2667.] = [2_667] = 1-x, 1-y, 2-z; [2567.] = [2_567] = -x, 1-y, 2-z; [2666] = 1-x, 1-y, 1-z; [2576] = -x, 2-y, 1-z. Cg2, Cg3 and Cg4 are centroids of C2–C7, C8–C13 and N5/C19–C23, respectively. Cg(I) = plane number I; Cg–Cg = distance between ring centroids (Ang.). Beta is the displacement angle between the ring normal of plane I and the centroid vector.

Table S6. Hydrogen bond and π–π interaction geometries (Å, °) in the $(SSZ)_2$·PHE cocrystal [a]

	Type	D-H···A	D-H	H···A	D···A	D-H···A	ARU (J)
1		O1-H1···N1	0.82	1.94	2.733(4)	163	[1555.02]
2	Intra	O3-H30···O2	0.82	1.85	2.575(4)	146	
3		N4-H40···N5	0.86	2.06	2.899(5)	164	[7656.01]
4		C3-H3···O2	0.93	2.48	3.354(5)	158	[1555.01]
5		C6-H6···O1	0.93	2.59	3.281(5)	132	[1555.01]
6	Intra	C18-H18···O5	0.93	2.48	2.872(5)	105	
7	Intra	C21-H21···O5	0.93	2.43	2.982(7)	118	
8		C22-H22···O3	0.93	2.59	3.379(6)	143	[3766.01]
9		C24-H24···O4	0.93	2.46	3.129(6)	129	[7656.01]
**Cg(I)**	**Cg(J)**	**Cg-Cg**	**Interplanar distance**		**Dihedral Angle**	**Beta**	**ARU (J)**
Cg2	Cg2	3.607(4)	3.4329(14)		0.72(16)	17.9	[2655.01]
Cg3	Cg5	4.168(4)	3.2938(16)		13.64(18)	25.1	[2655.02]
Cg3	Cg5	3.841(4)	3.5585(16)		13.64(18)	23.6	[2755.02]
Cg3	Cg6	4.168(4)	3.2938(16)		13.64(18)	25.1	[4465.02]
Cg3	Cg6	3.841(4)	3.5585(16)		13.64(18)	23.6	[4565.02]

[a] Symmetry codes: [3766.] = 2-x, 1-y, 1-z; [7656.] = 3/2-x, 1/2-y, 1-z; [2655.] = 1-x, y, 1/2-z; [2755.] = 2-x, y, 1/2-z; [2755.] = 2-x, y, 1/2-z; [4565] = x, 1-y, 1/2+z. Cg2, Cg3, Cg5 and Cg6 are centroids of C8–C13, C14–C19 and C1–C6, respectively. Cg(I) = plane number I; Cg–Cg = distance between ring centroids (Ang.). Beta is the displacement angle between the ring normal of plane I and the centroid vector.

Table S7. Hydrogen bond and π–π interaction geometries (Å, °) in the SSZ·4AP salt [a]

	Type	D-H···A	D-H	H···A	D···A	D-H···A	ARU (J)
1		N1-H1N···O2	0.86	2.15	2.878	142	[1555.01]
2		N2-H2A···O10	0.86	2.03	2.880	168	[1555.02]
3		N2-H2B···O5	0.86	2.17	3.031	178	[1654.01]
4		N3-H3N···O6	0.86	2.31	3.137	162	[2865.02]
5		N3-H3N···O7	0.86	2.59	3.155	124	[2865.02]
6	Intra	O3-H3O···O4	0.82	1.84	2.564	146	
7		N4-H4A···O10	0.86	2.02	2.812	153	[1555.02]
8		N4-H4B···O4	0.86	2.12	2.900	150	[2667.01]
9		N6-H6N···N5	0.86	2.08	2.926	167	[2576.01]
10	Intra	O8-H8O···O9	0.82	1.76	2.492	148	
11		N10-H10N···N9	0.86	2.05	2.894	166	[2855.02]
12		C3-H3···O9	0.93	2.39	3.314	170	[1555.02]
13		C7-H7···O9	0.93	2.59	3.105	116	[1655.02]
14		C9-H9···O5	0.93	2.27	3.137	155	[2667.01]
15	Intra	C11-H11···O2	0.93	2.47	3.029	119	
16		C14-H14···O1	0.93	2.44	3.110	129	[2576.01]
17	Intra	C17-H17···O2	0.93	2.58	2.923	103	
18	Intra	C29-H29···O7	0.93	2.44	3.004	119	

19		C32-H32···O6	0.93	2.47	3.141	129	[2855.02]
20	Intra	C35-H35···O7	0.93	2.54	2.916	104	

**Cg(I)**	**Cg(J)**	**Cg-Cg**	**Interplanar distance**	**Dihedral Angle**	**Beta**	**ARU (J)**
Cg2	Cg3	3.908(3)	3.731(2)	16.4(2)	33.7	[2577.01]
Cg2	Cg4	3.733(3)	3.512(2)	8.9(2)	17.9	[2766.02]
Cg3	Cg4	3.955(3)	3.549(2)	7.7(2)	33.8	[1466.02]
Cg5	Cg6	4.085(3)	3.655(2)	13.4(2)	39.8	[2765.02]

[a] Symmetry codes: [2576.] = -x, 2-y, 1-z; [2855.] = 3-x, -y, -z; [1655.] = 1+x, y, z; [1654.] = 1+x, y, -1+z; [2865.] = 3-x, 1-y, -z; [2667.] = 1-x, 1-y, 2-z; [2577.] = -x, 2-y, 2-z; [2766.] = 2-x, 1-y, 1-z; [1466.] = -1+x, 1+y, 1+z; [1644.] = 1+x, -1+y, -1+z; [2765.] = 2-x, 1-y, -z. Cg2, Cg3, Cg4, Cg5 and Cg6 are centroids of C16–C21, C22–C27, N9/C29–C33, C34–C39 and C40–C45, respectively. Cg(I) = plane number I; Cg–Cg = distance between ring centroids (Ang.). Beta is the displacement angle between the ring normal of plane I and the centroid vector.

Table S8. Hydrogen bond and π–π interaction geometries (Å, °) in the SSZ·TMD salt [a]

	Type	D-H···A	D-H	H···A	D···A	D-H···A	ARU (J)
1	Intra	O3-H03···O4	0.82	1.78	2.513(11)	147	
2		N2-H2···N1	0.86	2.09	2.920(13)	162	[2558.01]
3		N5-H5···O5	0.86	1.75	2.610(13)	175	[1555.01]
4		C1-H1···O2	0.93	2.42	3.124(14)	133	[2558.01]
5	Intra	C4-H4···O1	0.93	2.49	3.046(17)	119	
6	Intra	C6-H6···O1	0.93	2.51	2.900(16)	106	
7		C24-H24B···N4	0.97	2.54	3.458(17)	159	[2666.01]

Cg(I)	Cg(J)	Cg-Cg	Interplanar distance	Dihedral Angle	Beta	ARU (J)
2	3	3.964(11)	3.407(5)	5.2(6)	26.5	[2657.01]
4	4	4.104(11)	3.326(6)	0.0(7)	35.9	[2766.02]
5	5	3.647(13)	3.610(8)	0	8.1	[2865.02]

[a] Symmetry codes: [2558.] = -x, -y, 3-z; [2666.] = 1-x, 1-y,1-z; [2657.] = 1-x, -y, 2-z; [2766.] = 2-x, 1-y, 1-z. Cg2, Cg3, Cg4 and Cg5 are centroids of C6–C11, C12–C17, N5/C19–C23 and N6/C27–C31, respectively. Cg(I) = plane number I; Cg–Cg = distance between ring centroids (Ang.). Beta is the displacement angle between the ring normal of plane I and the centroid vector.

Table S9. Hydrogen bond and π–π interaction geometries (Å, °) in the SSZ·IMZ·MeCN salt solvate[a]

	Type	D-H···A	D-H	H···A	D···A	D-H···A	ARU (J)
1	Intra	O1-H1···O2	0.82	1.8	2.530(4)	147	
2		N1-H1A···O5	0.86	2.56	3.129(3)	125	[3667.01]
3		N1-H1A···N2	0.86	2.09	2.944(3)	170	[3667.01]
4		N5-H5A···O3	0.86	1.82	2.673(4)	174	[1556.01]
5		N6-H6···O2	0.86	1.86	2.672(3)	156	[2555.01]
6	Intra	C2-H2···O6	0.93	2.51	3.057(3)	118	
7		C5-H5···O5	0.93	2.46	3.085(3)	124	[3667.01]
8	Intra	C7-H7···O6	0.93	2.53	2.911(3)	105	
9		C100-H10B···N100	0.96	2.57	3.487(8)	160	[4565.03]
10		C100-H10C···O5	0.96	2.41	3.212(5)	141	[1555.01]
11		C21-H21···N100	0.93	2.57	3.365(6)	144	[1555.03]
12		C22-H22···O3	0.93	2.35	3.220(4)	155	[4565.01]

Cg(I)	Cg(J)	Cg-Cg	Interplanar distance	Dihedral Angle	Beta	ARU (J)
1	1	4.1552(17)	3.3891(11)	0.00(13)	35.3	[3666.01]
2	3	4.2756(17)	3.0805(10)	11.01(12)	33.8	[4555.01]

[a] Symmetry codes: [3667.] = 1-x, 1-y, 2-z; [4565.] = x, 3/2-y, 1/2+z; [1556.] = x, y, 1+z; [2555.] = -x, 1/2+y, 1/2-z; [3666.] = 1-x, 1-y, 1-z; [4555.] = x, 1/2-y, 1/2+z; [4554] = x, 1/2-y, -1/2+z. Cg1, Cg2 and Cg3 are centroids of N1/C1–C5, C6–C11 and C12–C17, respectively. Cg(I) = plane number I; Cg–Cg = distance between ring centroids (Ang.). Beta is the displacement angle between the ring normal of plane I and the centroid vector.

Table S10. Selected geometric parameters (Å, °)

Solid State	C1-N2	N2-S1	N1-C1-N2-S1	N2-S1-C2-C3
SSZ (imide form)	1.348 (4)	1.586 (3)	164.70	43.90
SSZ (amide form)	1.425 (2)	1.6539 (16)	107.13	86.38
$(SSZ)_2$·BPY·$(Tol)_{0.8}$	1.353 (2)	1.604 (2)	173.42	96.03
$(SSZ)_2$·BPE·$(EtOH)_2$	1.350 (4)	1.602 (2)	−173.66	73.70
$(SSZ)_2$·PHE	1.331 (5)	1.594 (3)	179.70	−117.89
SSZ·4AP	1.339 (5)	1.582 (4)	178.95	−74.11
SSZ·TMD	1.350 (2)	1.597 (8)	−178.26	78.38
SSZ·IMZ·MeCN	1.343 (3)	1.603 (2)	−178.45	56.11

Table S11. Summary of the various contact contributions to the SSZ Hirshfeld surface area in different cocrystals and salts

	**O-H**	**H-H**	**C-H**	**C-O**	**C-C**	**N-H**	**O-N**	**N-C**	**N-N**	**H-S**	**O-O**
$(SSZ)_2\cdot BPY\cdot (Tol)_{0.8}$	21.5	32.8	21.6	3.8	4.5	12.4	1.5	1.9	0	0	0
$(SSZ)_2\cdot BPE\cdot (EtOH)_2$	23	37.8	15.6	3.9	3.8	9.3	1.3	4.3	0.5	0	0.5
$(SSZ)_2\cdot PHE$	24.3	40.6	13.1	2.7	6.0	10.0	1.2	1.2	0.3	0	0.6
SSZ·4AP	21.7	27.5	23.4	3.6	6.5	9.7	2.9	2.2	1.7	0	0.8
SSZ·TMD	23.3	32.2	22.5	4.0	3.8	9.7	3.1	1.0	0.3	0	0
SSZ·IMZ·MeCN	25.0	27.1	22.2	3.2	4.9	12.1	1.0	4.4	0	0.1	0.1

REFERENCES

(1) Filip, L. A.; Caira, M. R.; Farcas, S. I.; Bojit, M. T., Triclinic polymorph of sulfasalazine. *Acta Cryst.* **2001,** C57, 435–436.

(2) Blake, A. J.; Lin, X.; Schroder, M.; Wilson, C.; Yuan, R. X., The imide tautomer of sulfasalazine. *Acta Crystallogr C.* **2004,** 60, o226-228.
